# Navigating the multifaceted intricacies of the Na^+^-Cl^−^ cotransporter, a highly regulated key effector in the control of hydromineral homeostasis

**DOI:** 10.1152/physrev.00027.2023

**Published:** 2024-02-08

**Authors:** A. V. Rioux, T. R. Nsimba-Batomene, S. Slimani, N. A. D. Bergeron, M. A. M. Gravel, S. V. Schreiber, M. J. Fiola, L. Haydock, A. P. Garneau, P. Isenring

**Affiliations:** ^1^Department of Medicine, Nephrology Research Group, Laval University, Quebec City, Quebec, Canada; ^2^Service de Néphrologie—Transplantation Rénale Adultes, Hôpital Necker-Enfants Malades, AP-HP, INSERM U1151, Université Paris Cité, Paris, France

**Keywords:** cation-Cl^−^ cotransporter, distal tubule, Gitelman syndrome, Na^+^-Cl^−^ cotransporter, SLC12A3

## Abstract

The Na^+^-Cl^−^ cotransporter (NCC; SLC12A3) is a highly regulated integral membrane protein that is known to exist as three splice variants in primates. Its primary role in the kidney is to mediate the cosymport of Na^+^ and Cl^−^ across the apical membrane of the distal convoluted tubule. Through this role and the involvement of other ion transport systems, NCC allows the systemic circulation to reclaim a fraction of the ultrafiltered Na^+^, K^+^, Cl^−^, and Mg^+^ loads in exchange for Ca^2+^ and $\mathrm{HC}O_{3}^{-}$. The physiological relevance of the Na^+^-Cl^−^ cotransport mechanism in humans is illustrated by several abnormalities that result from NCC inactivation through the administration of thiazides or in the setting of hereditary disorders. The purpose of the present review is to discuss the molecular mechanisms and overall roles of Na^+^-Cl^−^ cotransport as the main topics of interest. On reading the narrative proposed, one will realize that the knowledge gained in regard to these themes will continue to progress unrelentingly no matter how refined it has now become.

## Graphical abstract

**Figure:**
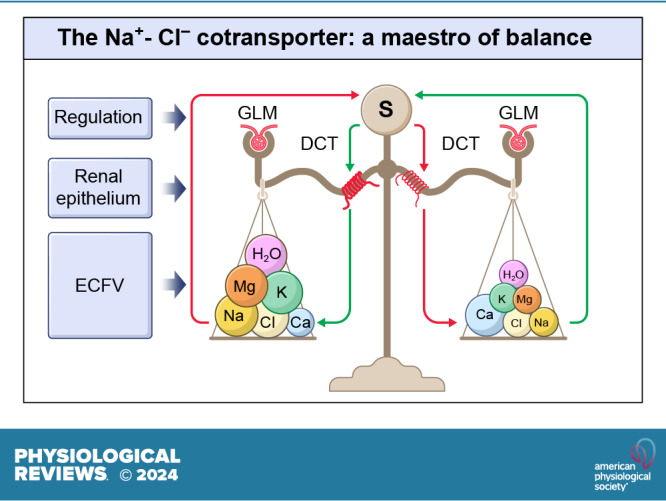

CLINICAL HIGHLIGHTS

- The Na^+^-Cl^−^ cotransporter (NCC) is responsible for the apical step of Na^+^ and Cl^−^ reabsorption by the distal convoluted tubule (DCT).
- When this mechanism is prevented through the administration of thiazides or in the setting of Gitelman syndrome, the extracellular fluid volume (ECFV) and K^+^ reabsorption by and beyond the DCT both decrease. When, by contrast, the same mechanism is accelerated in the setting of Gordon syndrome where WNK signaling is excessive, the ECFV and K^+^ reabsorption both increase. This gain of function in NCC is in fact exquisitely sensitive to thiazides.
- Other conditions can also cause the Na^+^-Cl^−^ cotransport mechanism to accelerate. One of these is primary hyperaldosteronism, whereby an increase in NCC activity leads to ECFV expansion while minimizing renal K^+^ secretion. Under such circumstances, thiazides could be detrimental by preventing this NCC-dependent K^+^-sparing effect from taking place. SGLT2 inhibition is an additional NCC-stimulating condition, as the apical Ca^2+^-sensing receptor of the DCT appears to react to glucose by eliciting WNK activity. Under such circumstances, thiazides could also be detrimental by preventing NCC from compensating for the loss of ions that comes with osmotic diuresis.
- Otherwise, the Na^+^-Cl^−^ cotransport mechanism is a target of choice for the treatment of essential hypertension.

## 1. INTRODUCTION

The Na^+^-Cl^−^ cotransporter (NCC; SLC12A3) is a member of the cation-Cl^−^ cotransporter (CCC) family (1, 2). Within this family, it belongs to a phylogenetic subgroup that comprises Na^+^-K^+^-Cl^−^ cotransporter 1 (NKCC1; SLC12A2) and Na^+^-K^+^-Cl^−^ cotransporter 2 (NKCC2; SLC12A1). In primates, NCC also comes as three different splice variants called NCC1, NCC2, and NCC3, and in *Equus* species, it comes as two isoforms called NCCα (SLC12A3) and NCCβ (SLC12A10).

The main function of NCC is to mediate the cotranslocation of Na^+^ and Cl^−^ across the apical membrane of the distal convoluted tubule (DCT) (3). This transport mechanism allows 5% of the ultrafiltered NaCl load to be reabsorbed by the kidney and affects the handling of other ions (most notably of Ca^2+^, Mg^2+^, and K^+^) in the middle portion of the distal nephron (3–6). By achieving such roles, NCC is also involved in extracellular fluid volume (ECFV) regulation, K^+^ homeostasis, and urinary dilution as a consequence (6–9).

The Na^+^-Cl^−^ cotransport mechanism was uncovered almost 50 years ago and attributed to a protein that was termed “NCC” 20 years later. It was linked early on to Gitelman syndrome and suspected of being an important determinant of blood pressure (BP) in the general population. It eventually became the object of great enthusiasm when two members of the WNK kinase family were found to be associated with a thiazide-sensitive form of familial hypertension and to coordinate the tubular handling of various ions in the face of perturbed K^+^ or ECFV balance.

More recently, a number of advances have led to a significant leap forward in our understanding of how the role of NCC is integrated with that of the other DCT-based ion transport systems and how the Na^+^-Cl^−^ cotransport mechanism is structurally orchestrated. These advances came in particular from the identification of several DCTopathy-associated genes (>15) and from the acquisition of high-resolution structural data for all of the ion-transporting CCCs.

The present review aims to describe the molecular mechanisms and dynamics of NCC under both normal and pathological conditions. It also aims to present the progress made, unresolved issues, and future avenues in the field of Na^+^-Cl^−^ cotransport along with personal views on the models and concepts proposed. As will be seen, the identification of NCC has led the way to many discoveries and a remarkable comprehension of how the DCT carries out its various functions.

## 2. CHARACTERIZATION OF Na^+^-Cl^−^ COTRANSPORT IN THE PREMOLECULAR ERA

### 2.1. Prequel to the Identification of NCC

NCC is inhibited by a group of drugs that come under the umbrella of thiazides (10) and that were derived initially from a molecule called benzothiadiazine. They consist of biheterocyclic benzenes in which one of the cycles contains a sulfur and two nitrogen atoms (11, 12). They played a key role in the identification of the Na^+^-Cl^−^ cotransport mechanism some 40 years ago and became the “modern diuretics” along with the loop and K^+^-sparing agents after the early 1960s (13).

Renal diuretics have been in use for over 5,000 years. Initially, they included inorganic mercury salts, xanthine derivatives such as caffeine, and osmotic salts or sugars (14–16). During the sixteenth century, mercury salts were medically advocated for the treatment of edematous states even though they were associated with serious side effects and of limited efficacy (17, 18). After World War II, organic mercurials and acetazolamide were introduced but did not prove to offer added benefits (19–21).

During the 1950s, a biochemist (James Sprague) from the Merck Sharp & Dohme Research Laboratories pioneered the thiazides and thus reached a key milestone in doing so (12). The first drug synthesized among these diuretics was termed chlorothiazide and marketed under the name “Diuril” in 1958. Chlorothiazide and following thiazide-type diuretics rapidly gained in popularity afterward, as they were also shown to be unexpectedly efficacious in the treatment of high blood pressure (22).

Shortly after their discovery, and based on a combination of approaches that included renal micropuncture/microperfusion studies in particular, thiazides were found to exert their diuretic action mainly by inhibiting renal salt reabsorption at the level of the DCT (9, 23–25). However, their precise functional target in this nephron segment was not identified until 30 years later, that is, around the mid-1980s.

In this review, the term “premolecular era” will refer to a period during which NCC was characterized before its molecular identity was formally uncovered. This era lasted ∼20 years, in that the Na^+^-Cl^−^ cotransport mechanism was described for the first time in a fish species during the mid-1970s and the protein at play isolated from the exact same species by expression cloning around the mid-1990s.

### 2.2. Identification of NCC

It was Renfro (26, 27) who first identified this mechanism, and he did so more specifically in the urinary bladder epithelium of winter flounder, a flatfish known also as *Pseudopleuronectes americanus*. The transport moiety uncovered then was seen to be apically based and to allow for net ion uptake in the absence of K^+^. It was also described as one in which the absorption of Na^+^ and Cl^−^ were coupled, occurred at equal rates, and required the presence of both ions.

Soon after, Stokes (28) found that this transport moiety in winter flounder bladder could be inhibited by thiazides according to Michaelian kinetics. In the years that followed, many investigators raised the possibility that an analogous carrier system would be found in the DCT of mammalian species and that it would likely be the main target of thiazides. During the mid-1980s, renal microperfusion/micropuncture studies in rat DCT allowed Costanzo (29) to confirm this possibility and additional investigators (30–32) to eventually come to similar conclusions.

In early 1980, another ATP-independent cotransport mechanism was reported by Geck et al. (33) in Ehrlich–Lettre ascites cells. It was also found to allow for the electroneutral comovement of Na^+^ and Cl^−^ but had the particularity of also requiring K^+^, of operating with four transport sites (1 for Na^+^, 1 for K^+^, and 2 for Cl^−^), and of being furosemide sensitive. On the basis of these findings, the K^+^-dependent and K^+^-independent systems were predicted at that time to be mediated by different sets of proteins.

### 2.3. Localization

**FIGURE 1.**
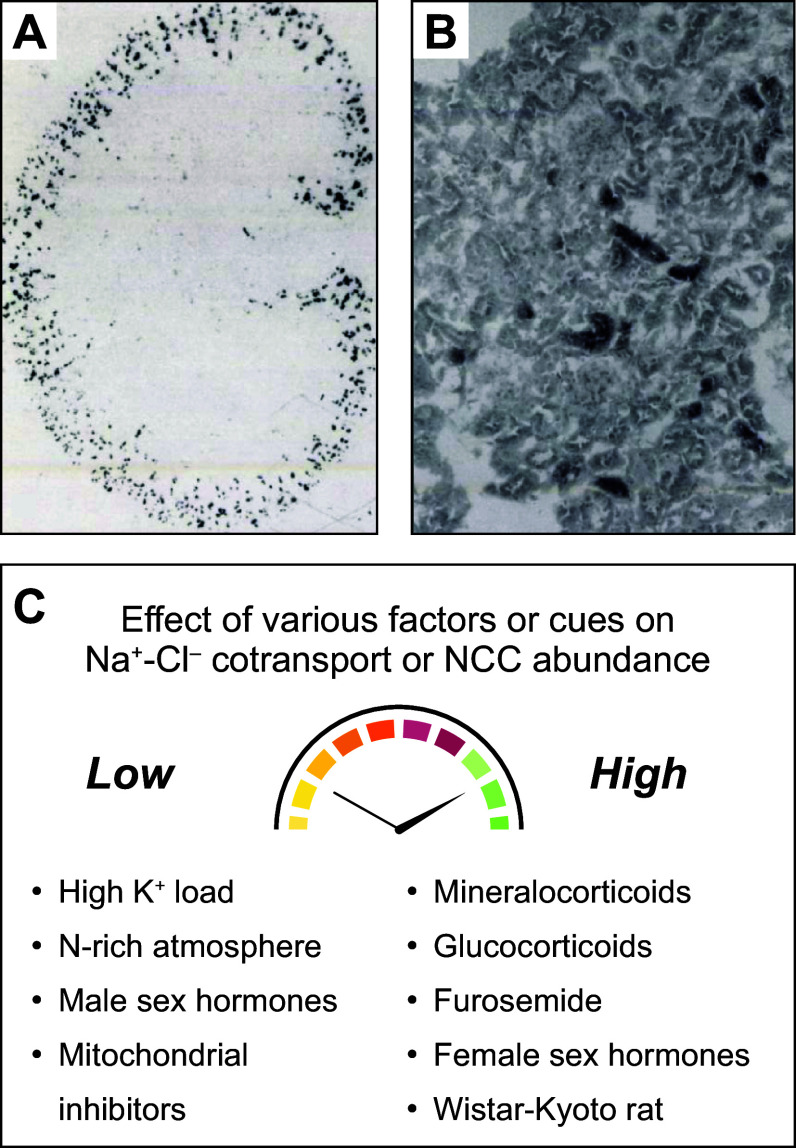
Functional characterization of the Na^+^-Cl^−^ cotransporter (NCC) during the premolecular era. *A*: autoradiogram of a slide-mounted rat kidney section prelabeled with [^3^H]metolazone (MTZ). Signal intensity was considered a marker of NCC abundance and/or Cl^−^ binding by the cotransporter. *B*: same as *A* except that the coverslip was examined at higher resolution under oil immersion. *C*: effects of various conditions, states, factors, or interventions on Na^+^-Cl^−^ cotransport or on NCC abundance. The data shown were mostly from micropuncture/microperfusion studies and [^3^H]MTZ binding assays. The images displayed in *A* and *B* are from Beaumont et al. in *Journal of Pharmacology and Experimental Therapeutics* (35). They are reproduced in this figure with permission by the copyright holders.

The renal micropuncture/microperfusion studies carried out initially provided robust evidence that NCC was expressed in the DCT most predominantly. Just before the 1990s, Beaumont et al. (34) provided additional evidence in this regard by obtaining autoradiographic images of [^3^H]metolazone (MTZ)-labeled rat, mouse, and rabbit kidney sections [see examples in Fig. 1, A and B, from Beaumont et al. (35)]. They found a pattern of signals that was consistent with selective localization of the isotope to the luminal side of the DCT. Of note, the labeling observed was nonetheless uneven in intensity among cell types in this nephron segment.

Still during the 1980s, rabbit blastocysts and gallbladder epitheliocytes were also both found to express a loop diuretic-resistant, K^+^-independent Na^+^-Cl^−^ cotransport system that did not appear to result from the combined activities of Na^+^/H^+^ and Cl^−^/$\mathrm{HC}O_{3}^{-}$ exchangers (36–38). At that time, however, the sensitivity of this system to thiazides was not reported for either cell type. Before the cloning of NCC, there was thus no evidence for or against the existence of NCC outside of the kidney and urinary tract.

### 2.4. Functional Properties

#### 2.4.1. Methods exploited.

Before the molecular era, two approaches were exploited to establish the functional properties of the Na^+^-Cl^−^ cotransport mechanism. One such approach consisted of transport studies in micropunctured/microperfused DCT and in bladder membranes isolated from winter flounder. The other approach consisted of binding experiments in which the substrate used corresponded to rat membranes derived from renal cortex and the ligand to [^3^H]MTZ (see FIGURE 1, *A* AND *B*).

The binding studies were also exploited to gain insight into the features of ion transport by NCC. They showed, for instance, that [^3^H]MTZ retention in membranes correlated inversely with extracellular (o) [Cl^−^] ([Cl^−^]_o_) or luminal (lu) [Cl^−^] ([Cl^−^]_lu_) and positively with[Na^+^]_o_ or [Na^+^]_lu_, suggesting that Cl^−^ and thiazides competed for the same site and that Na^+^ transport facilitated thiazide binding. As such, an increase in [^3^H]MTZ retention would indicate decreased Cl^−^ transport and increased Na^+^ transport, whereas decreased [^3^H]MTZ retention would indicate the opposite.

#### 2.4.2. Stoichiometry of ion transport.

The earliest experiments of Renfro (26, 27) and Stokes (28) consisted of actual ion transport studies in which NCC activity was measured through Na^+^ and Cl^−^ fluxes (*J*) across the apical membrane of perfused winter flounder bladder. As stated, the movement of Na^+^ and Cl^−^ by NCC was found then to be inwardly directed, quantitatively coupled, and unaffected by changes in voltage. These findings were thus consistent with an ion transport stoichiometry of 1 Na^+^:1 Cl^−^. Subsequent experiments in micropunctured/microperfused rat and rabbit DCT led to similar deductions (31).

#### 2.4.3. Orientation of ion movement and symmetry.

For different reasons, the cosymport mechanism identified in winter flounder bladder and in rodent DCT had already been predicted early on to be naturally involved in net ion reabsorption. In particular, it was found to be apically localized, to be unaffected by membrane potential (*E*_m_) as an electroneutral ion transport system, and to be Na^+^ dependent, whereas intracellular [Na^+^] ([Na^+^]_i_) in both these tissues is usually lower than [Na^+^]_lu_.

One could have still wondered whether [Na^+^] in the lumen of a rodent DCT would have been a limiting factor to ion absorption by NCC. However, this was not seen to be the case. Based on renal micropuncture/microperfusion studies, for instance, [Na^+^]_lu_ at the entry of this nephron segment usually ranges between 40 and 80 mM even in NaCl-depleted subjects (4, 31, 39, 40), and when it is set at 40 mM ion influx rates by the cotransporter are already at or near saturation (31, 40).

Most carriers are able to operate in both directions. The renal micropuncture/microperfusion carried out during the premolecular era confirmed that the same held true for NCC (31). They showed more specifically that ion influx by NCC increased progressively between 0 and 100 [Na^+^]_lu_ or between 0 and 100 [Cl^−^]_lu_ (while maintaining the counterion at 100 mM) but that $J_{\mathrm{(N}a^{+})}$ and $J_{\mathrm{(C}l^{-})}$ were both close to 0 at ∼10 mM and negative below this concentration.

As to whether or not ion influx and efflux by the cotransport mechanism are kinetically symmetrical, this question does not appear to have been addressed during the premolecular era despite its potential relevance from a physiological perspective. If, for instance, NCC was globally less efficient in the outward mode, this would allow for higher levels of NaCl absorption along the DCT by failing to act as a (strong) secretory pathway in the later portion of this nephron segment, where [Na^+^]_lu_ and [Cl^−^]_lu_ are lower than in the initial portion.

#### 2.4.4. Substrate affinity.

The studies of Stokes (28) in winter flounder bladder had included ion transport measurements as a function of [Na^+^]_lu_ and [Cl^−^]_lu_ to determine the apparent affinity of NCC for the transported ions. Based on the data shown, both $K_{\mathrm{m(N}a^{+})}$ and $K_{\mathrm{m(C}l^{-})}$ appeared to lie between 5 and 10 mM. In microperfused rat DCT, Velázquez et al. (31, 40) subsequently found that the affinity for the ions was once again in the vicinity of 10 mM and that NCC would thus operate below saturation in this nephron segment.

Experiments were also conducted in the 1980s to determine the affinity of NCC for thiazides. In the case of hydrochlorothiazide (HCTZ), *K*_i_ values were in the low nanomolar range based on transport assays in winter flounder (28) and ∼100 nM based on [^3^H]MTZ binding assays in isolated rat renal membranes (34). The binding assays showed additionally that *K*_i_ varied extensively (from 3,700 to 0.4 nM) among thiazides as follows (from lowest to highest): methyclothiazide > polythiazide > MTZ > indapamide > chlorthalidone > HCTZ > trichlormethiazide > chlorothiazide (34).

#### 2.4.5. Ion dependence of ion transport and of thiazide binding.

Still during the premolecular era, Beaumont et al. (34) and Tran et al. (41) were the first research group to report a detailed kinetic portrait of ion transport and diuretic binding by NCC. They were in fact the investigators who found that MTZ binding increased with [Na^+^]_o_ and decreased with [Cl^−^]_o_. They concluded from their observations that NCC contained two high-affinity binding sites, one for Na^+^ and one for Cl^−^ or thiazides, that Cl^−^ competed with MTZ for the same site, that binding of Na^+^ to the Na^+^ binding site decreased $K_{\mathrm{m(C}l^{-})}$, and that unloading of Na^+^ produced the opposite effect. These findings were consistent with a model of ordered ion binding.

#### 2.4.6. Specificity of ion binding sites.

In the same studies, Beaumont et al. (34) and Tran et al. (41) further found that Br^−^ and I^−^ decreased [^3^H]MTZ binding substantially as if they were able to act as strong competitors of the drug for occupancy of the Cl^−^ binding site. Interestingly, Li^+^, K^+^, Rb^+^, and $NH_{4}^{+}$ were also seen to increase [^3^H]MTZ binding in the higher concentration ranges but only slightly so and much less so than Na^+^ (41), suggesting then that they could act as weak competitors of Na^+^ for occupancy of the Na^+^ binding site.

During the 1980s and 1990s, there were no reports on whether NCC had the ability of translocating Li^+^, K^+^, Rb^+^, and/or $NH_{4}^{+}$ through the Na^+^ transport site and of translocating Br^−^ and/or I^−^ through the Cl^−^ transport site. Demonstrating that it had this ability would have required influx or uptake studies to be carried out in which these potential surrogates were added to the external medium in replacement of Na^+^ or Cl^−^ and their cellular accumulation monitored through direct measurement assays.

Based on such studies for NKCC1, it was found that this other protein could act not only as a Na^+^-K^+^-Cl^−^ cotransporter but also as a Li^+^-K^+^-Cl^−^, Na^+^-Rb^+^-Cl^−^, and Na^+^-$NH_{4}^{+}$-Cl^−^ cotransporter (42, 43). Showing that NCC could have acted analogously as a Li^+^-Cl^−^, K^+^-Cl^−^, and/or Rb^+^-Cl^−^ cotransporter would have certainly been of interest. In particular, it could have provided insight into the phylogeny of the ion binding sites in the CCCs and the mechanisms of Li^+^ handling by the renal epithelium. It is of note that even if the Na^+^-Cl^−^ cotransport mechanism is considered K^+^ independent, it is not completely abolished in the absence of [Na^+^]_o_ (Isenring et al., personal observations).

### 2.5. Effects of Various Cues on NCC Activity

Before NCC was cloned, its activity or abundance at the cell surface was known even then to be affected by a variety of cues or factors (see FIGURE 1, *A*–*C*). Much of the knowledge gathered in this regard was once more obtained through renal micropuncture/microperfusion and [^3^H]MTZ binding studies. Even if it was based in part on indirect measurements, it laid the foundation for many concepts that are considered well established in the current era.

One cue that was found initially to affect NCC activity is the response elicited by steroid hormones. In Sprague-Dawley rats, for instance, Na^+^ absorption and [^3^H]MTZ binding by the DCT were shown to decrease after orchiectomy or adrenalectomy, to increase after administration of corticosteroids (including both mineralocorticoids and glucocorticoids), and to be higher in females (44–47). These observations led to the conclusion that the DCT contributed to the antinatriuretic and kaliuretic effect of steroid hormones through the involvement of NCC.

Chen et al. (48) also found that NCC activity and/or abundance was sensitive to furosemide and HCTZ. In particular, these investigators observed that renal slices obtained from rats after acute or chronic administration of either drug exhibited a substantial increase in [^3^H]MTZ binding by the DCT. Whereas furosemide could have led to this outcome by increasing NaCl delivery to NCC-expressing cells, HCTZ was seen to exert the same reaction for reasons that were unclear in those days.

Another cue that was seen to affect NCC activity is the response elicited by high K^+^ intakes. Through micropuncture/microperfusion studies, this response was found more specifically to be associated with increased K^+^ secretion just beyond the DCT (49, 50) and decreased HCTZ-induced natriuresis by interfering with Na^+^-Cl^−^ cotransport (49, 51). It was hypothesized then that the observed reduction in NCC activity was responsible for the kaliuretic effect of K^+^ loading by allowing higher levels of Na^+^ delivery to the middle and later portion of the distal nephron (49).

In the late 1980s, Beaumont et al. (35) identified additional NCC-affecting cues. By way of illustration, rat renal sections incubated with mitochondrial inhibitors or in a nitrogen-rich atmosphere were seen to exhibit lower levels of [^3^H]MTZ labeling. Of interest, this outcome was not seen to occur in membrane preparations, such that it was presumably accounted for by an intracellular or circulating factor “x.”

Renal sections examined by Beaumont et al. (52) in another study were found additionally to exhibit higher levels of [^3^H]MTZ labeling when they were obtained from spontaneously hypertensive Wistar-Kyoto rats, indicating perhaps that NCC contributed to the development of high BP in the animal model exploited. This possibility was consistent with the observation that the hypertensive model was known to be typically fluid overloaded early in its life (53–55).

### 2.6. Physiological Roles

As mentioned above, thiazides were found just after their discovery to inhibit NaCl reabsorption by the DCT. They were also found to be effective agents in the treatment of high BP, especially in that of the low-renin form of essential hypertension (56, 57). They were thus suspected early on to act on a transport mechanism that played an important role in BP control by impacting on ECFV secondarily (52, 57, 58).

It has long been recognized that thiazides increase Ca^2+^ absorption by NCC-expressing epithelia such as the DCT (29, 59) and bladder of winter flounder (60). They were said at one point to do so by stimulating the cellular uptake of Ca^2+^ through an electrogenic pathway on the apical side (61), as they also caused NCC-expressing cells to hyperpolarize, i.e., to reset *E*_m_ at higher levels (62–64). Whether this effect of thiazides was an indication that NCC is involved in Ca^2+^ handling was apparently not explored at that time.

As also mentioned above, the Na^+^-Cl**^−^** cotransport mechanism was attributed the additional role of maintaining K^+^ balance (65). For instance, a high K^+^ load was found to decrease NaCl reabsorption by the DCT and this decrease to induce K^+^ secretion by the same nephron segment and beyond. The appropriate homeostatic response under such circumstances was indeed for kaliuresis to increase, and NCC reacted accordingly and independently of ECFV.

## 3. MOLECULAR IDENTIFICATION OF NCC

### 3.1. Cloning of NCC

#### 3.1.1. Species.

##### 3.1.1.1. winter flounder.

The protein sequence of NCC was discovered by Gamba et al. (1) just before the mid-1990s. The strategy used was to isolate mRNA from the bladder of 80 winter flounders and test size-divided fractions of the material obtained for their ability to induce increased Cl^−^-dependent, MTZ-sensitive ^22^Na**^+^** influx in *Xenopus laevis* oocytes. A cDNA library was ultimately generated out of one of the most promising RNA fractions and pool-screened with the same assay until the transport signal of interest could be narrowed to single clones.

One of the clones isolated was subjected to further functional analyses in the same expression system. It was found to cause cell swelling under isotonic condition but not in the presence of MTZ. The increase in ^22^Na^+^ uptake was also seen to be inhibited by several thiazides (polythiazide > MTZ > cyclothiazide > HCTZ > chlorothiazide), to be insensitive to 5-(*N*-ethyl-*N*-isopropyl)amiloride (EIPA), furosemide, amiloride, and acetazolamide, and to vary as a function of [Na^+^]_o_ and [Cl^−^]_o_ based on a one-binding site model. From these studies, $K_{\mathrm{m(N}a^{+})}$ was also reported to be 25 mM and $K_{\mathrm{m(C}l^{-})}$ 14 mM.

As for the nucleotide sequence of the cDNA cloned (L11615 in GenBank), it was predicted to code for a 1,023-amino acid protein comprised of a 12-transmembrane domain (TMD) segment flanked on both sides by long cytoplasmic domains rich in putative phosphorylation sites. The translation product was also predicted to harbor two N-X-S/T glycosylation sites within the fourth extracellular loop (EL) of the membrane domain and confirmed to be processed as a glycoprotein based on in vitro translation studies in pancreatic microsomes (1).

The protein identified in winter flounder was named thiazide-sensitive cotransporter (TSC) to begin with. However, it is now more commonly referred to as NCC given that the other CCCs are also acronymized based on their transport function and that loop diuretics and some of the thiazides can act on other carrier systems. Eventually, TSC/NCC was named SLC12A3 based on the HUGO nomenclature. In addition to being poorly evocative, this SLC12A3 abbreviation is also a low-yield MeSH term to search for the older publications on the Na^+^-Cl**^−^** cotransport mechanism. As such, the term “NCC” will continue to be used throughout the remainder of the present review.

##### 3.1.1.2. rat.

After their initial cloning efforts, Gamba et al. (66) tried to determine whether the K^+^-independent Na^+^-Cl**^−^** cotransport mechanism identified in rat and mouse DCT could be ascribed to an ortholog of winter flounder NCC (flNCC). The approach used initially was to carry out low-stringency Northern blot analyses in which mRNA extracts from rat and mouse renal cortex were exposed to flNCC-derived riboprobes. Three bands varying between 3.8 and 5.5 kb in size were identified.

Soon after, the residue sequence of rat NCC (rtNCC) was the inaugural mammalian ortholog to be identified. The strategy used was to screen a size-selected rat renal cortex cDNA library with NCC-derived riboprobes. In a first step, the probe used was from flNCC and hybridized with the library at low stringency, and in a second step it was from the 5′ end of rtNCC (identified through the first step) and hybridized with the same library at high stringency. In the end, full-length cDNA clones of interest were isolated.

**FIGURE 2.**
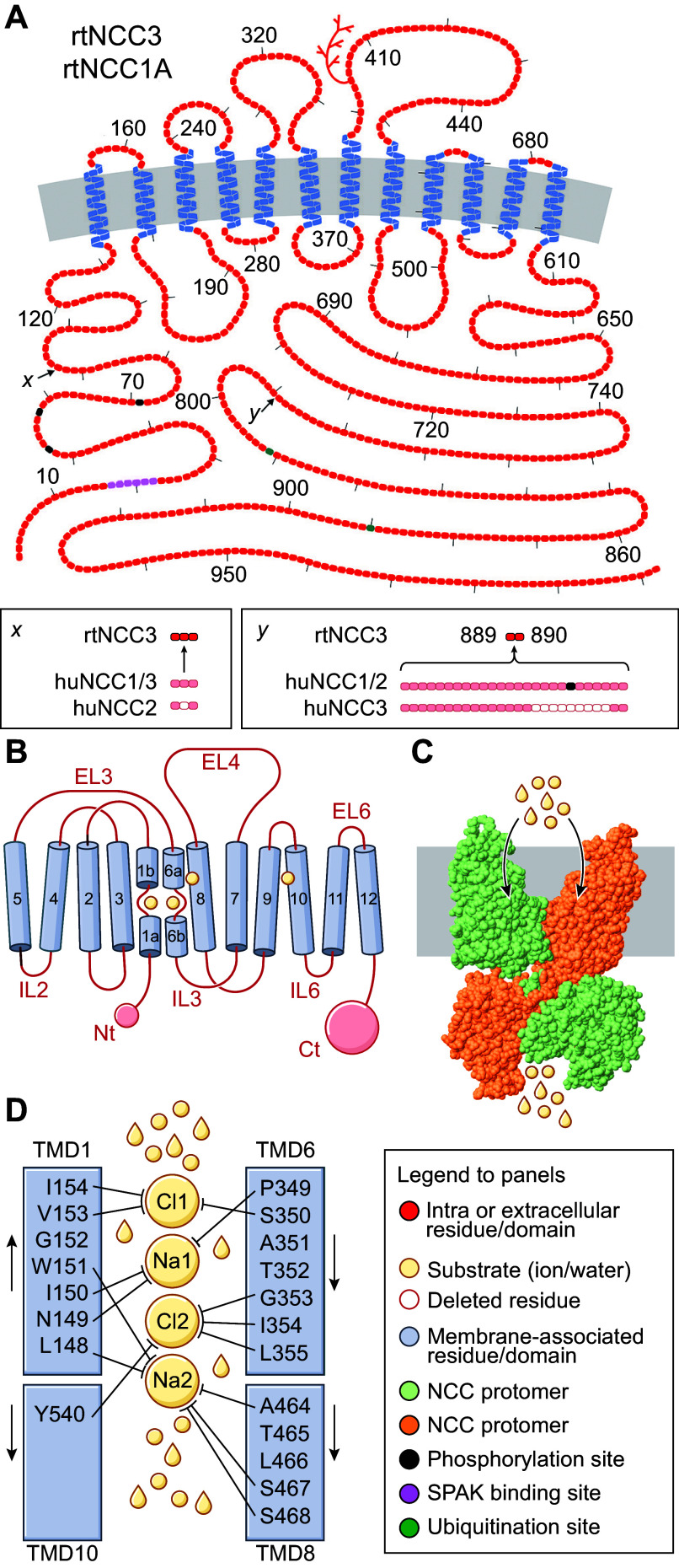
Structural feature of the Na^+^-Cl^−^ cotransporter (NCC). *A*: hydropathy plot model of rat (rt)NCC3/rtNCC1A. Residues are shown as round or square forms (1 form per residue) and the glycosylation site of rtNCC3/rtNCC1A as a branched line. *x* and *y* show how rtNCC3/rtNCC1A differ from the human variants (huNCC1/huNCC1B, huNCC2/huNCC3C, and huNCC3/huNCC1C) in residue composition. Color code used is indicated at *right* in *D*. The model was drawn using the program PLOT. *B–D*: hydropathy plot model, structure, and ion binding sites of huNCC based on cryo-EM determinations. Color code used is indicated at *right* in *D*. Ct, COOH terminus; EL, extracellular loop; IL, intracellular loop; Nt, NH_2_ terminus; TMD, transmembrane domain. See glossary for additional abbreviations.

These clones included two cDNAs splice variants that corresponded to duplicates of one another except that one missed 231 bp in the 3′ untranslated region (UTR) (GenBank accession number of longest clone is U10097.2). As for the translation product (see FIGURE 2*A*), it was predicted to consist of a 1,002-residue polypeptide and found to share 63% identity and 83% similarity in amino acid composition with flNCC as well as the same structural traits except for having a single putative glycosylation site in the fourth EL. As for flNCC, in vitro translation studies confirmed that rtNCC was also a glycoprotein.

The functional characteristics of rtNCC were examined once again in the *Xenopus laevis* oocyte expression system (66). They were found to be very similar to those of flNCC except that HCTZ was seen to be a more potent inhibitor than cyclothiazide to reduce Na^+^-Cl**^−^** cotransport. Studies were carried out in addition to demonstrate that ^22^Na**^+^** uptake was both K**^+^** independent and bumetanide insensitive. However, it was only in a subsequent publication that the kinetic characteristics of ion transport by rtNCC were reported.

##### 3.1.1.3. human.

After the residue sequence of rtNCC was uncovered, that of the human ortholog came to be known in 1996 through additional cloning efforts by two independent research groups. It was searched for by one of the groups (67) to determine whether it was a candidate gene for Gitelman syndrome and obtained from a human genome cosmid library with human NCC (huNCC)-derived cDNA probes. It was isolated by the other group (68) from a human kidney cDNA library with rtNCC-derived cDNA probes and mapped to chromosome 16q13 by fluorescence in situ hybridization.

### 3.2. CCC Family

#### 3.2.1. Family members.

The residue sequences of NCC, NKCC1, and NKCC2 were discovered almost contemporaneously. Back in the mid-1990s, all three isoforms were expected to share homology with another ion carrier that was known by function to sustain an electroneutral Na^+^-independent K^+^-Cl^−^ cotransport mechanism. It was only a few years later that not one but four distinct ion carriers were found to account for this other mechanism and to be indeed phylogenetically related with the other carrier systems. They were called K^+^-Cl^−^ cotransporter (KCC)1, KCC2, KCC3, and KCC4 (69–72).

The term “CCC” is now used as the name of the family to which NCC, the NKCCs, and the KCCs belong (2). These proteins have in common that they all mediate the electroneutral movement of Cl^−^ along with that of a cation, i.e., of Na^+^ and/or K^+^. Two other CCC isoforms were uncovered in the early 2000s and called CCC8 (SLC12A9) and CCC9 (SLC12A8). As it stands, however, they appear to be involved in polyamine and amino acid transport rather than in cation-Cl^−^ cotransport (73).

**FIGURE 3.**
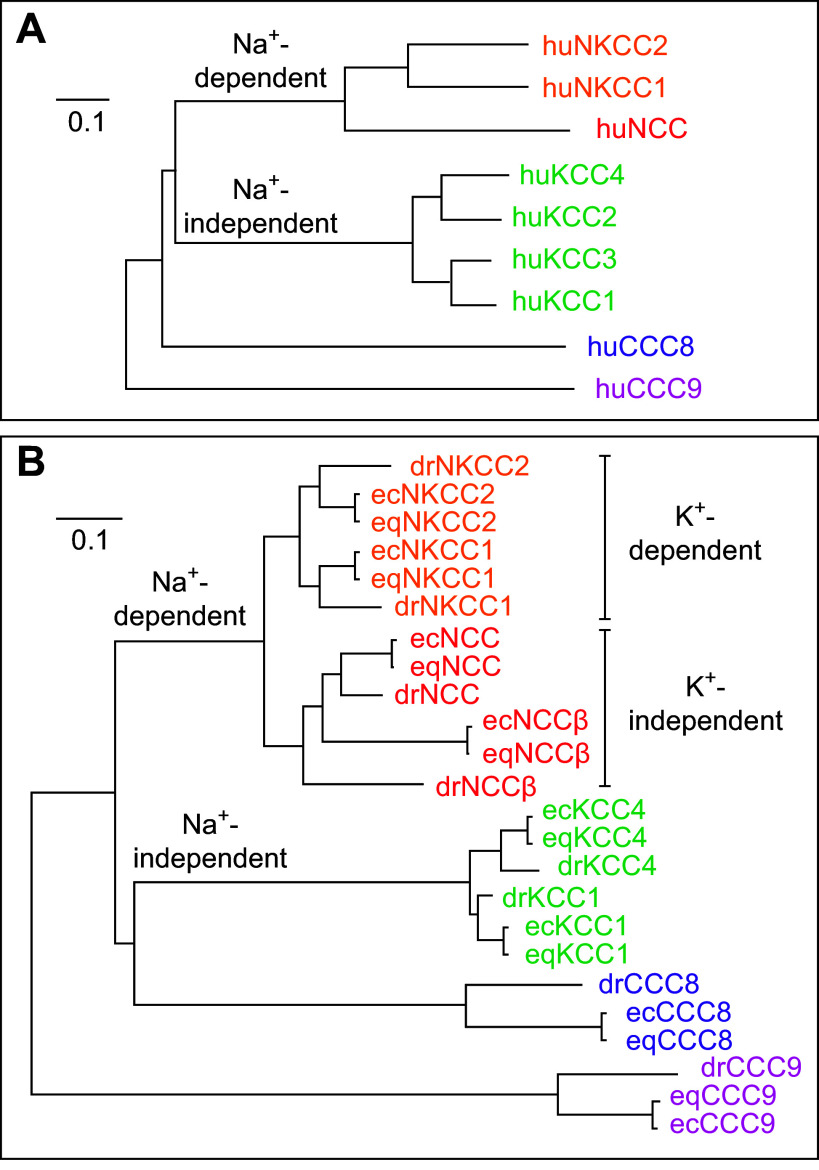
Phylogenetic analyses. *A*: phylogram of functionally active human cation-Cl^−^ cotransporter (huCCC) isoforms. Na^+^-Cl^−^ cotransporter (NCC)β (also called SLC12A10, NCC2, or CCC10) is thus excluded. Sequences used: NKCC1, NP_001037.1; NKCC2, NP_000329.2; NCC, NP_000330.2; K^+^-Cl^−^ cotransporter (KCC)1, NP_005063.1; KCC2, NP_001128243.1; KCC3, NP_598408.1; KCC4, NP_006589.2; CCC8, NP_064631.2; and CCC9, NP_078904.3. *B*: phylogram of functionally active *Danio rerio* (dr)CCC, *Equus caballus* (ec)CCC, and *Equus quagga* (eq)CCC isoforms. NCCβ is thus included. Sequences used: drNKCC1, NP_001002080.1; drNKCC2, XP_021323409.1; drNCC, NP_001038545.1; drKCC1, XP_691291.2; drKCC4, XP_696060.6; drCCC8, NP_001122020.1; drCCC9, XP_005167859.1; drNCCβ, NP_001154850.2; ecNKCC1, XP_005599514.2; ecNKCC2, XP_003363557.1; ecNCC, XP_014593920.2; ecKCC1, XP_001498498.1; ecKCC4, XP_023481655.1; ecCCC8, XP_001505118.1; ecCCC9, XP_023479371.1; ecNCCβ, LC727702.1; eqNKCC1, XP_046522358.1; eqNKCC2, XP_046510184.1; eqNCC, XP_046540395.1; eqKCC1, XP_046538230.1; eqKCC4, XP_046527564.1; eqCCC8, XP_046521243.1; eqCCC9, XP_046514771.1; and eqNCCβ, XM_046655358.1. The trees were constructed with the programs Clustal Omega and FigTree, using the longest residue sequences for each of the CCCs. In each panel, the scale corresponds to a genetic distance.

As shown in FIGURE 3*A*, the nine CCC isoforms can be subdivided into four different groups based on homology sharing in amino acid composition. Whereas NCC falls in one of these groups along with NKCC1 and NCC, the KCCs fall in another group. As for CCC8 and CCC9, they each belong to an individual clade in which they are the sole representative. They are also as far from one another as they are from the Na^+^-dependent or Na^+^-independent CCCs.

Based again on the phylogram of FIGURE 3*A*, it is interesting to observe that the acquisition or loss of a strong Na^+^ transport site has apparently occurred in a common but distant predecessor within the family and that it is associated with diverging paths between the Na^+^-dependent and Na^+^-independent CCCs. As ancestor proteins are generally seen as generalists that can recognize a broad set of ligands (74, 75), one could hypothesize that the more primitive CCCs actually consisted of Na^+^-K^+^-Cl^−^ cotransporters.

The CCCs form a group of ion transport systems that are subsumed under a much larger mantle that has become to be known as the amino acid-polyamine-organocation (APC) superfamily. Non-CCC members within this larger group also act as solute carriers, but they are not Na^+^ coupled when their substrates consist of amino acids. A functionally active Na^+^ binding site could have thus been acquired very early on in the APC lineage.

Bacterial APCs have been used for quite some time as tractable models of solute carriers to decipher the functional and structural properties of mammalian derivatives. The characterization of two such APCs (AdiC and ApcT) has been particularly informative and has even allowed pioneering concepts to be established. Whereas the former is expressed in different strains of *Escherichia coli* and acts as an arginine/agmatine exchanger to enable survival in acidic environments (76), the latter is expressed in thermophilic bacteria/archaea and acts as an amino acid-H^+^ symporter (77).

#### 3.2.2. A new era.

The cloning of NCC almost 30 years ago was a prelude to much progress in our understanding of how this transporter operates, is regulated, and affects solute homeostasis. It led to such progress by allowing many experimental tools to be developed such as NCC-specific DNA, RNA, and antibody probes, NCC-based cDNA constructs, and NCC-null mouse models. These tools made it possible, for instance, to study NCC through structure-function analyses in expression systems, localization assays in animal tissues, and genetic approaches in both human and animal models.

It is of note that the heterologous expression system used to characterize the molecular and functional properties of wild-type (WT) and mutant NCC has been limited almost entirely to the oocyte of *Xenopus laevis*. Although this expression system was exploited in the absence of more convenient alternatives, it still permitted milestones to be reached, all the more so that it is known for bioprocessing many foreign proteins into native-form structures (78–80). However, it does come with some limitations, as discussed below.

## 4. CHARACTERIZATION OF NCC DURING THE MOLECULAR ERA

### 4.1. Identification of Orthologs and Isoforms

Following the identification of the protein responsible for the Na^+^-Cl^−^ cotransport mechanism before the mid-1990s and the advent of large-scale DNA sequencing projects a few years later, the residue sequence of >100 NCC orthologs came to be known. Whereas, interestingly, most mammals were found to express only one isoform of this carrier system, several fish species (including winter flounder) and some mammals were also found to express an additional isoform that can be spliced into a high number of variants (81, 82). This isoform is now referred to as NCCβ or SLC12A10 based on the HUGO nomenclature.

The cotransporter initially known as SLC12A3 was also termed NCCα by some investigators to distinguish it from NCCβ. It would have been perhaps preferable to use the acronym NCC2 and rename NCC to NCC1 in accordance with the taxonomic canvas that had been used to denominate the other CCC isoforms. Under this canvas, the number refers to a unique gene locus and the order of discovery and a letter after the number (e.g., NKCC2A, NKCC2B, etc.) to a splice variant. Given that the terms “NCC1” and “NCC2” are now used in another context (see below), SLC12A10 will still be called NCCβ/SLC12A10 in this review and SLC12A3 will continue to be called NCC.

Based on synteny and phylogenetic analyses, NCCβ/SLC12A10 has been found to be expressed more specifically in ray-finned teleosts, coelacanths, amphibians, reptiles, and a few mammals such as monotremes (e.g., platypus), horse, and zebra (82). It has also been found to have undergone deletion or pseudogenization in birds, a majority of mammals, and a few fishes, suggesting that it began to lose functionality in an ancestral species of extant aves. Surprisingly, this isoform is thiazide insensitive and apparently electrogenic (83–85), even though it shares high homology with SLC12A3, e.g., 79% in zebrafish.

FIGURE 3*B* is used to illustrate the phylogeny of NCCβ/SLC12A10 alongside the other CCC isoforms for three selected and apparently active orthologs. It is seen in particular that the 10 isoforms can still be subdivided into four different groups and that NCCβ/SLC12A10 falls in the K^+^-independent group of Na^+^-dependent CCCs. It is also seen that the distance between the mammalian NCCβ/SLC12A10s and mammalian NCCs is about the same as between the nonmammalian NCCβ/SLC12A10s and nonmammalian NCCs.

### 4.2. Identification of Splice Variants

The 3′-truncated rtNCC sequence reported by Gamba et al. (66) just before the mid-1990s was the object of no further characterization. As it stands, it is not possible to determine whether this clone was an actual splice variant, given that its nucleotide sequence does not appear to have been deposited in GenBank or described so as to understand which region of the 3′ UTR segment was missing. However, it appears that the 3′ UTR of NCC is represented by a number of splice variants based on blast searches of the EST databanks.

As schematized in FIGURE 2*A, x* AND *y*, huNCC comes as three functional splice variants (86, 87). Two such variants (termed NCC1 and NCC2) are comprised of 1,030 and 1,029 residues, respectively, are primate specific, and correspond to the sequence previously identified by Simon et al. (67). The other variant (NCC3) comprises 1,021 residues and corresponds to the sequences identified earlier on by Mastroianni et al. (68) in human kidney and Gamba et al. (66) in rat DCT. NCC1 and NCC2 have been collectively referred to as NCCsv (where “sv” stands for splice variant), as they are experimentally indistinguishable from each other.

The nomenclature used to designate huNCC splice variants is again not in accordance with the formerly exploited taxonomy and is unsatisfactory for at least three reasons. First, it generates confusion in that the number after the acronym refers to a splice variant and no longer to a gene locus. Second, this nomenclature does not respect the order of discoveries in that the residue sequence of rtNCC3 was reported before that of huNCC1 and huNCC2. Third, huNCC1 and huNCC2 are regrouped under the vocable huNCC_SV_ whereas huNCC3 is also a splice variant. A more congruent appellation scheme for huNCC3, huNCC1, and huNCC2 would have consisted of huNCC1A, huNCC1B, and huNCC1C, respectively.

The other differences among the variants are illustrated again in FIGURE 2*A*. In particular, the shortest cotransporter (huNCC3/huNCC1A) is identical to the longest one (huNCC1/huNCC1B) except for lacking nine residues in exon 20 from the use of an alternative 5′ donor splice site at the end of this exon (86, 87). As for the 1,029-residue cotransporter (huNCC2/huNCC1C), it is also identical to huNCC1/huNCC1B except for lacking E_95_ in the NH_2_ terminus (Nt) from the use of an alternative 3′ acceptor site at the beginning of exon 2 (86, 87). The term “NCC1-NCC2 (NCC1B-NCC1C)” is used here to designate these two splice variants.

Thus far, the cotransporter to have been the subject of most characterizations in heterologous expression systems is rtNCC3/rtNCC1A. Yet, and as also discussed below, one of the residues missing from exon 20 in this variant is now known to play an important role in carrier regulation (86, 87). Additionally, it is unclear that rtNCC3/rtNCC1A can serve as a good model of the same variant in other species given that exon 20 in rodent is 17 nucleotides shorter than that in huNCC1/huNCC1B and 16 shorter that in flNCC1/flNCC1B.

### 4.3. Localization of NCC

#### 4.3.1. Localization of NCC in the kidney.

Northern blot analyses carried out by Gamba et al. (1) showed that flNCC was expressed in winter flounder bladder. However, no signals could be seen when the RNA tested was from whole kidney in this species. One explanation could be that the message of interest failed detection as it was too dilute from the presence of medullary-derived mRNA. Another perhaps less likely explanation (see below) could also be that Na^+^-Cl^−^ cotransport in winter flounder DCT is mediated for the most part by NCCβ/SLC12A10.

Through a combination of qPCR, in situ hybridization, and immunodetection studies, NCC (SLC12A3) has now been found in the DCT of several freshwater, euryhaline, or seawater teleosts including mefugu, torafugu, Japanese eel, and zebrafish (88–90). Through more sensitive detection studies such as RNA-seq analyses, NCC has also been found in the kidney (presumably DCT) of several other teleosts including swamp eel, golden mahseer, Nile tilapia, turbot, Northern pike, Asian arowana, and spotted gar (91–94).

During the molecular era, Gamba et al. (66) were the first investigators to carry out localization studies of NCC in the kidney. With a combination of high-stringency Northern blot and in situ hybridization analyses, they confirmed that NCC was DCT specific in rat kidney and also found that it was in fact kidney specific as well. Other investigators eventually came to similar conclusions through various detection methods (95–98).

NCC was the object of immunodetection studies during the 1990s to determine its microanatomical and subcellular distribution along the renal epithelium of rat (99–102). Through this approach, it was found in two different studies to be localized at the apical membrane of DCT1 and DCT2 exclusively and in another study to be present at the same locations but with some extension into the connecting tubule (CNT). Additional immunodetection studies in mouse kidney subsequently revealed a similar pattern of expression (97, 103).

Tutakhel et al. (87) have more recently looked at the expression of the NCC variants in human kidney. Interestingly, they found slightly higher levels of NCC3/NCC1A- than of NCC1-NCC2/NCC1B-NCC1C-encoding mRNAs. They also detected the proteins translated from these mRNAs in the apical membrane of the DCT as well as in urinary extracellular vesicles. In the experiments carried out, no mention was made as to whether the variants exhibited differential distributions along the DCT. One might have expected such distributions to be in fact varied given that the cell population of this nephron segment is very heterogeneous in morphology and function.

#### 4.3.2. Localization of NCC outside of the DCT.

As stated above, NCC was already suspected of being expressed outside of the DCT during the premolecular era, that is, in the gallbladder and blastocysts of lagomorphs (104–106). Northern blot analyses conducted in the early molecular era showed that this cotransporter was in fact widely distributed in winter flounder, as it was detected in gonads, small intestine, skeletal muscle, eye, and brain among other tissues (1). However, and as also stated above, additional analyses conducted soon after in other vertebrates showed that NCC was apparently restricted to kidney (66).

In more recent years, the use of additional detection strategies and the advent of large-scale RNA and protein databanks have revealed that NCC is present in a multitude of cell types and tissues including various nephron segments (88). This finding is not necessarily unexpected given that RNA or protein detection methods have become exquisitely more sensitive than 20–30 years ago. In particular, they have allowed us to show that promoter leakage is a common trait among expressible genes such that the distribution of many proteins is much wider than initially documented.

NCC abundance in most tissues and cell types is still seen to be very low compared with renal cortex, yet one cannot conclude that Na^+^-Cl^−^ cotransport outside of the DCT would be too low to be of physiological relevance. In this regard, promoter leakage is considered by some investigators to be a neglected determinant of phenotype even if it is usually not associated with robust gene expression (107). As will be seen below, there are two tissues of interest in which local NCC activity could play an important functional role.

In mammals, NCC expression levels have not been found to be low in all cell types outside of the DCT. In particular, they are seen to be substantial in bladder and testis, in several types of cancerous tissues (e.g., renal, urothelial, and endometrial carcinomas), in a variety of glandular cells (e.g., gastric parietal and breast cuboidal cells), and in a number of immortalized cell lines (including most notably HeLa cells). However, the role that NCC might play in these other cell types is unclear.

### 4.4. Functional Characterization of Ion Transport NCC in Animal Species

#### 4.4.1. Ion affinities.

The affinities of NCC for the transported ions, those of flNCC (in winter flounder bladder and in *Xenopus laevis* oocytes) and those of rtNCC (in rat DCT), have already been reported above (28, 40). Those for rtNCC and mouse NCC (msNCC) were also determined later on but in the oocyte expression system exclusively. In the case of rtNCC, the values available were from several different experiments (108–112), and in the case of msNCC, they are from a single experiment (113).

A summary of the kinetic parameters obtained is shown in TABLE 1. It is seen that $K_{\mathrm{m(N}a^{+})}$ and $K_{\mathrm{m(C}l^{-})}$ for flNCC in bladder, for rtNCC in DCT and oocytes, and for msNCC in oocytes all lie between 5 and 10 mM. It is seen on the other hand that $K_{\mathrm{m(N}a^{+})}$ values for flNCC in oocytes are approximately threefold higher compared with these values and that $K_{\mathrm{m(C}l^{-})}$ values for flNCC in oocytes are approximately twofold higher. In all of the experiments conducted, transport activity came to subsaturation at [Na^+^]_o_ and [Cl^−^]_o_ of <30–40 mM.

**Table 1.** Summary of the kinetic parameters

Species	Cell Type or Tissue	$K_{m({\text{Na}}^{+})}$, mM	$K_{m({\text{Cl}}^{-})}$, mM	Relative Affinities for Thiazides (highest to lowest)
flNCC	Winter flounder bladder	5-10	5-10	PTZ ≅ MTZ > CTLD > HCTZ > TCMZ
	*Xenopus*	25-30	15-20	PTZ ≅ MTZ > TCMZ > HCTZ > CTLD
rtNCC	DCT	10	10	
	*Xenopus*	7	6	MTZ > TCMZ > HCTZ > PTZ > CTLD
msNCC	*Xenopus*	7	6	

CTLD, chlorthalidone; DCT, distal convoluted tubule; fl, winter flounder; HCTZ, hydrochlorothiazide; *K*_m_, ion constant; ms, mouse; MTZ, metolazone; NCC, Na^+^-Cl^−^ cotransporter; PTZ, polythiazide; rt, rat; TCMZ, trichlormethiazide.

It is of note that the *K*_m_ values of TABLE 1 were all derived from the Michaelis–Menten equation and with the assumption thus that Hill coefficients would be unitary at all times and that transport activities at 0 [Na^+^]_o_ ($V_{\mathrm{0(N}a^{+})}$) or 0 [Cl^−^]_o_ ($V_{\mathrm{0(C}l^{-})}$) would also be null at all times (108, 109, 111, 112). Yet it appears that many of the activity vs. concentration curves shown in the studies reported did not fit the data adequately by eye. For most of the CCCs, additionally, $V_{\mathrm{0(N}a^{+})}$, $V_{\mathrm{0(}K^{+})}$, and $V_{\mathrm{0(C}l^{-})}$ are never really zero, based on other studies and personal observations by Isenring et al. (2, 114–119). It is as such likely that a four-parameter equation (where Hill coefficients and *V*_0_ are also variables) would have yielded different *K*_m_ values.

Despite some issues as to how they were derived, the kinetic parameters reported for both rtNCC and msNCC were seen nonetheless to be functionally aligned with the chemical environment to which NCC is exposed along the apical side of the DCT (4, 31, 39, 40). In this environment, Na^+^ and Cl^−^ are indeed present at much lower concentrations than in serum (3-fold to 4-fold lower) and therefore require high-affinity transport systems for their transepithelial reabsorption to be significant and sustained (4, 31, 39, 40).

#### 4.4.2. Stoichiometry.

As a reminder, the Na^+^-Cl^−^ cosymport mechanism was found soon after its discovery to be electrically silent, allow for equal rates of translocation between the ions, and vary with [Na^+^]_o/lu_ and [Cl^−^]_o/lu_ according to Hill coefficients of 1.0. These findings implied that the movement of one Na^+^ was coupled with that of one Cl^−^ (28, 120, 121). However, Hill coefficients in ion transport studies point to a minimal number of binding sites. As such, it is not possible to rule out that the stoichiometric rapport of substrate movement by NCC would be, for instance, 2 Na^+^:2 Cl^−^ per translocation cycle.

On the basis of kinetic studies discussed above, the dependence of heterologously expressed flNCC, rtNCC, and msNCC on [Na^+^]_o_ and [Cl^−^]_o_ was said to be best described with Hill coefficients of 1.0 (28, 40, 108–113). As mentioned, however, this parameter does not appear to have been iterated in the experiments carried out, and the activity vs. ion concentration curves derived from the equation used were not subjected to fit statistics. In addition, NCC was not characterized in *Xenopus laevis* oocytes through electrophysiological measurements to confirm that its activity was voltage insensitive.

Up until recently, a popular claim in the field of Na^+^-Cl^−^ cotransport was that the protein at play enclosed only two ion binding sites, one for Na^+^ and one for Cl^−^. However, this claim was not shared by all investigators, in that the number of apparent Cl^−^ transport sites in the NKCCs, which are highly homologous to NCC, was higher than “1.0” and that it had also been found to vary as a function of [Cl^−^]_o_ (2, 114, 116–118). Previous inferences based on Hill coefficient determinations are now known to be invalid given that all of the ion-transporting CCCs were recently shown by cryo-EM studies to harbor at least two binding sites for Cl^−^ (122).

In our opinion, the question that pertains to the number of ion binding sites in NCC is not trivial. If, for instance, NCC was able to transport 3 Na^+^:3 Cl^−^ per translocation cycle, fine-tuning of its activity could then be most efficiently achieved through a change in conformation rather than in cell surface expression. If, along the same line, NCC was able to transport 1 Na^+^:2 Cl^−^ or 3 Na^+^:2 Cl^−^ under certain circumstances (there is currently no evidence against this possibility), it would then act as an electrogenic rather than electroneutral transport system and would affect (and be affected by) the ion transport systems with which it is coexpressed quite differently.

#### 4.4.3. Ion dependence and model of ion binding.

Monroy et al. (108) conducted additional experiments in the late 1990s and early 2000s to gain granular insight into the kinetic characteristics of ion transport by rtNCC in *Xenopus laevis* oocytes. The strategy used was to compute the *K*_m_s and *V*_max_ of Na^+^ transport at a fixed concentration of [Na^+^]_o_ or of [Cl^−^]_o_ but at increasing concentration of the counterion and to interpret the data obtained in light of the “rapid equilibrium approach” of Segel (123). Vázquez et al. (112) repeated these experiments in 2001 for flNCC.

Through this strategy, the authors observed for both orthologs inverse correlations between $K_{\mathrm{m(C}l^{-})}$ and [Na^+^]_o_ and between $K_{\mathrm{m(N}a^{+})}$ and [Cl^−^]_o_ but positive correlations between *V*_max_ and [Na^+^]_o_ or [Cl^−^]_o_. They concluded that the transport of Na^+^ by NCC was dependent on that of Cl^−^ and the transport of Cl^−^ dependent on that of Na^+^. After analyzing the data based on the rapid equilibrium approach, Monroy et al. (108) concluded in the end that ion binding to NCC was random as opposed to ordered. The main argument behind this claim was that *V*_max_ for Na^+^ transport should not have been affected by both [Na^+^]_o_ and [Cl^−^]_o_ in a model where Na^+^ binds first and is unloaded first.

It should be remembered that Tran et al. (41) and Beaumont et al. (34) had previously shown that $K_{\mathrm{m(N}a^{+})}$ for NCC did not vary inversely as a function of [Cl^−^]_o_ in rat renal membranes. The data of these two research groups had also been exploited afterward by Chang and Fujita (124) to propose a model of ion binding that was ordered in appearance. As such, the results and predictions of Monroy et al. (108) were partly inconsistent with those of other investigators in the field.

Aside from the issues raised in regard to the approach used for data fitting, another reason to question the validity of the random model is that ion transport by NKCC1 in duck red blood cells was shown during the late 1990s to be ordered and to occur in a first-on first-off sequence scheme (125). If the random model did apply to NCC, one would then wonder why and how two carriers that are so homologous to each other would have evolved so as to exhibit such divergent translocation mechanisms.

**FIGURE 4.**
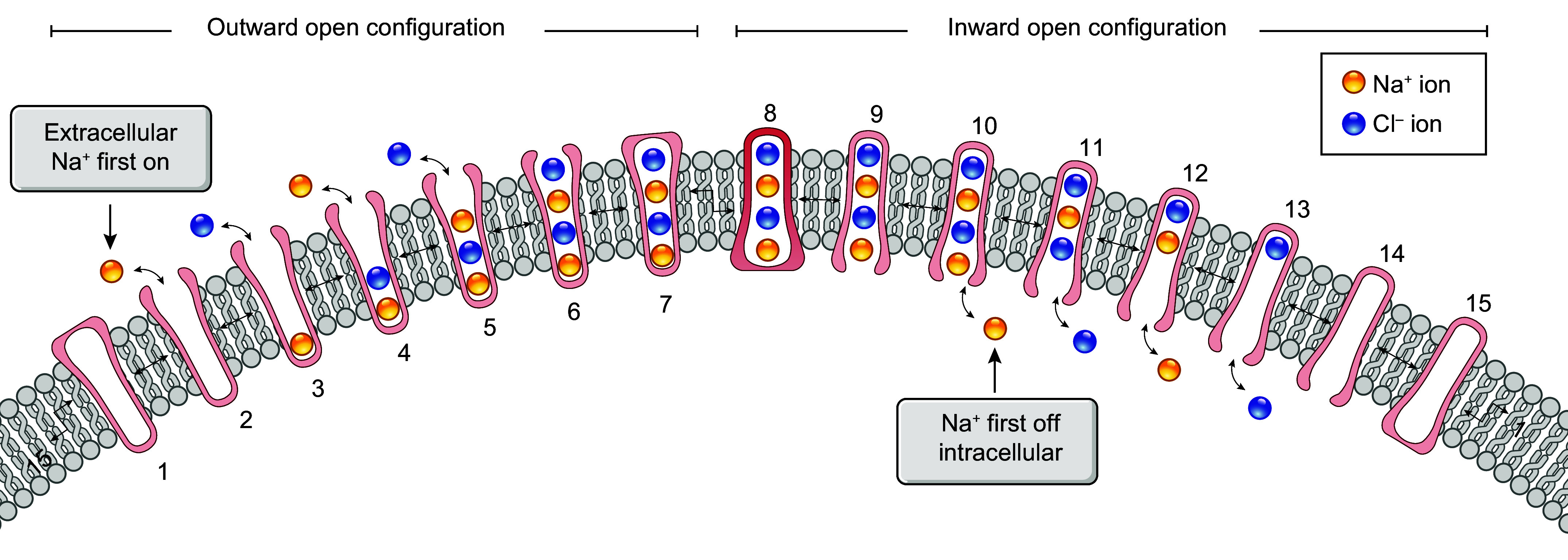
Proposed model of ion binding by the Na^+^-Cl^−^ cotransporter (NCC). In the scheme presented, the transport cycle involves 4 ion binding sites (2 for Na^+^ and 2 for Cl^−^) and is based on ordered binding and glide symmetry where first on is first off. This scheme is supported by indirect evidence through [^3^H]metolazone (MTZ) binding assays and the characterization of NKCC1 (34, 41, 125). The model depicted can be summarized as follows. When the carrier is empty, it adopts an outward-open configuration (*1*) and undergoes disocclusion (*2*) to be loaded from the extracellular side by 4 ions (Na1, Cl1, Na2, and Cl2) in successive steps (*3–6*). Once it is loaded, the carrier becomes occluded momentarily (*7*), adopts an inward-open configuration (*8*), and undergoes disocclusion once again (*9*) to release all 4 ions internally in the same order (*10–13*). When the carrier is no longer occupied by ions (*14*), it undergoes another episode of occlusion (*15*) and is translocated back to the outward-open configuration (*1*).

Collectively, the evidence supporting the idea that the transport cycles of both carriers proceed according to an ordered-binding, glide symmetry scenario (34, 41, 124, 125) remains stronger than the evidence supporting the absence of predictable reactions (108, 112). In FIGURE 4, we propose a hypothetical model of ion binding by NCC in the light of the most likely scheme. The model presented is also based on a four-ion binding site cotransport system (2 for Na^+^ and 2 for Cl^−^) to integrate the cryo-EM findings in regard to the number of substrates that can occupy the translocation pathway during a transport cycle.

#### 4.4.4. Orientation of ion movement and specificity of ion binding sites.

The question of whether the kinetics of ion binding, translocation, and dissociation are equal in both the influx and efflux modes has not been addressed during the molecular era, either. The same holds true in regard to the transportability of surrogate substrates such as Li^+^, K^+^, Rb^+^, $NH_{4}^{+}$, and Br^−^. For reasons mentioned above, these questions remain to be explored and the experiments required to address them would be easy to conduct.

One of the ions that was shown to interfere with MTZ binding during the premolecular era was I^−^. Transport studies carried out more recently showed that this halide ion could permeate NCC and do so as efficiently as Cl^−^ (126). It is now also known that ^131^I^−^ can be used in ion transport assays as a more convenient tracer than ^22^Na^+^ to measure Na^+^-Cl^−^ cotransport. Based on these observations, one must further consider the possibility that NCC could be involved in I^−^ uptake by certain cell types and, accordingly, play an important role in iodine/iodide homeostasis.

Another substrate that has been suspected of permeating the CCCs is water (127–130). Interestingly and yet intriguingly, the movement of this other substrate was found additionally to be ion coupled. As such, it was hypothesized that in the case of NKCC1 water transport by this carrier could occur against unfavorable osmotic gradients and allow the Na^+^-K^+^-Cl^−^ cotransport mechanism to induce faster rates of RVI responses and ion movement than if it was water impermeable.

As discussed below, cryo-EM-based structural determinations have recently led to the identification of water-interacting residues in the solvent-accessible vestibule of the Na^+^-dependent CCCs and have shown that these residues could coordinate the binding of ions at nearby sites. At this point in time, however, a role for NKCC1 in direct water transport is still uncertain and will require demonstration through additional approaches.

#### 4.4.5. Effect of changes in pH on NCC activity.

An interesting study by Fanestil et al. (131) showed that renal sections obtained from Wistar-Kyoto rats exhibited lower levels of [^3^H]MTZ labeling under a $NH_{4}^{+}$-Cl^−^-rich diet before euthanasia and higher levels of labeling under a Na^+^-$\mathrm{HC}O_{3}^{-}$-rich diet. These observations could have suggested that the activity of NCC in these models was pH dependent. However, it was not possible to determine whether the effects seen resulted from a change in intracellular or luminal free H^+^ concentration and whether other factors such as change in Na^+^ or Cl^−^ delivery to the DCT could have been at play.

Based on prior studies, those of Monroy et al. (108) and Vázquez et al. (112) on the functional properties of rtNCC and flNCC in *Xenopus laevis* oocytes, carrier activity was found in contrast to be unaffected by pH_o_ within the 6.0–8.0 unit range. However, the plasma membrane of this cell type is virtually impermeable to protons (132, 133), such that substantial acidification or alkalinization of the cytoplasm does not occur when pH_o_ is varied.

It is of note that many of the other ion-transporting CCCs have been shown to be pH_i_ sensitive (133). It is thus tempting to postulate that the same holds true for NCC, all the more so given that the observations of Fanestil et al. were potentially consistent with this possibility. If NCC was upregulated by alkalemia and downregulated by acidemia through an intracellular site, it could then be considered as one of the players that account for the effects of changes in systemic pH on salt handling by the nephron.

#### 4.4.6. Residues involved in ion binding.

##### 4.4.6.1. lessons from the nkccs.

###### 4.4.6.1.1. The chimera approach.

Isenring et al. (119, 134–136) were the first research group to search for ion transport sites in a CCC. They had used a mutagenic approach in which residues or domains were interchanged between NKCC1 orthologs (human and shark) that were known to exhibit marked differences in their affinities for the transported ions. The goal of these studies was to identify residues/domains that specified such differences and that could thus be involved in ion coordination. The expression system used then consisted of HEK-293 cells.

The chimera-based approach exploited showed in essence that TMD2, TMD4, and TMD7 contained most if not all of the molecular determinants that accounted for the differences in ion affinities between human NKCC1 (huNKCC1) and shark NKCC1 (saNKCC1). However, the TMDs at play differed as a function of the ion transported. In particular, TMD2 and TMD7 appeared most important in determining Na^+^ affinity, TMD2 and TMD4 Rb^+^ affinity, and TMD2, TMD4, and TMD7 Cl^−^ affinity. A number of candidate residues within these TMDs had also been identified.

NKCC2 was found during the 1990s to exist as three splice variants that were called NKCC2A, NKCC2B, and NKCC2F. Although, interestingly, these variants were found to be nearly identical to one another except for eight residues in TMD2 and three residues in the following intracellular loop (IL1), they were also seen to exhibit marked differences in their affinities for the transported ions. They were thus exploited in structure-function studies to determine which of the divergent residues accounted for the functional differences (114, 137–140).

The experiments conducted with this other isoform were also based on a chimera approach, but the expression system exploited consisted of *Xenopus laevis* oocytes. The data obtained allowed them to find that whereas TMD2 was key in specifying Na^+^, Rb^+^, and Cl^−^ affinity, IL1 was by contrast key in specifying Cl^−^ affinity at one of the Cl^−^ binding sites. These data were thus consistent with those of the previous studies on the huNKCC1-saNKCC1 chimeras in highlighting the importance of TMD2 as an affinity-modifying domain.

###### 4.4.6.1.2. Scanning mutagenesis.

More recently, Somasekharan et al. (141) used an approach called cysteine- and tryptophan-scanning mutagenesis to determine whether TMD3 in huNKCC1 was involved in substrate binding, as was known to be the case for the corresponding domain in the APC family members AdiC and ApcT. Single-point mutants were thus generated by replacing each residue in the α-helix with a Tyr or Cys residue. In these studies, the Tyr residue was used as replacement to mimic the ion- or furosemide-bound state and the Cys residue to probe the physical area surrounding TMD3 based on the effect of membrane-impermeant methanethiosulfonate reagents.

Several mutations were seen to produce large changes in ion affinities, ion translocation rates, or loop diuretic affinity. They were all localized on the same side of TMD3 and predicted thus to face the solvent-accessible vestibule. On the basis of the data obtained, residues toward the intracellular end of TMD3 were also predicted to be part of the translocation pathway in an inward-open conformation and M_382_ to be part of an extracellular gate against ion and diuretic entry into the vestibule.

#### 4.4.7. Residues involved in ion transport for NCC.

In the mid-2000s, Moreno et al. (110) exploited the chimera-based approach of Isenring et al. (119, 134–136) to identify affinity-specifying residues/domains in NCC. The transporters used then consisted of rtNCC and flNCC, as both were known to exhibit differences in kinetic characteristics, and the expression system *of Xenopus laevis* oocytes once again. These studies showed that the difference in Na^+^ affinity could be attributed to variant residues in all of the TMDs and the difference in Cl^−^ affinity to variant residues in TMD1 to TMD7 exclusively.

Interestingly, a naturally occurring and potentially relevant polymorphism in the TMD4 of huNCC (G264A) had been previously identified by Melander et al. (142) during the course of linkage analyses with BP variations in the general population. This polymorphism was shown later on to increase the diuretic action of furosemide in vivo, as if it led NCC to be less effective in handling a higher salt load (143), and to cause intrinsic cotransporter activity to decrease but Cl^−^ affinity to increase in vitro (109).

Be that as it may, many of the affinity-modifying domains identified in NCC had not been found to act as such in NKCC1. These findings might have thus come as a surprise given that the Na^+^-dependent CCCs share very similar functional characteristics and are highly homologous in residue sequence, especially across the membrane-attached domain. As argued below, there are various explanations that could account for what could appear as apparent discrepancies.

One simple explanation pertains to the limitations that come with the use of chimera-based approaches in structure-function studies. In particular, residues that are important in defining functional characteristics will be missed if they are the same between orthologous forms of a transporter. In the last five TMDs, for instance, the percentage of identical residues between huNKCC1 and saNKCC1 is higher than it is between rtNCC and flNCC, i.e., 90% versus 85%, respectively.

If ion binding to NCC was actually random rather than ordered as in the case of the NKCCs, another explanation would rest on this distinctive peculiarity. One must remember that in a multi-ion carrier system, binding of the first on is usually the dependent parameter (119, 125, 134–136, 144, 145). If, let us say, Na^+^ binds to NCC before Cl^−^ in 50% of the transport cycles instead of 100%, a change in $K_{\mathrm{m(C}l^{-})}$ resulting from a residue substitution in a Cl^−^ transport site would then be associated with a change in $K_{\mathrm{m(N}a^{+})}$ for these cycles only. As such, the averaged $K_{\mathrm{m(N}a^{+})}$ under this scenario would be much less affected than in an ordered Na^+^-first scheme.

Otherwise, the mutations identified in Gitelman syndrome have provided rather limited insight into the molecular determinants through which NCC handles the ions during translocation. There are two reasons behind this shortcoming. First, many of these mutations were found to be very disruptive and/or to result in premature degradation of the carrier. Second, most if not all of these mutations have still not been tested by cryo-EM to understand their structural impact.

### 4.5. Pharmacological Inhibition by Ion Transport Inhibitors

#### 4.5.1. Thiazides.

##### 4.5.1.1. potency as a function of molecules and species.

The initial functional characterization of flNCC and rtNCC in *Xenopus laevis* oocytes included affinity measurements for various thiazides (1, 66). The results of these studies have already been presented in sect. 3.1. The profiles seen were in fact quite similar between the two orthologs, but the overall affinities for any given thiazide tended to be lower for flNCC (TABLE 1).

The two carriers were eventually characterized through several additional studies in the same expression system to assess the inhibitory potency of additional thiazides (108–113). It was found that the affinity of either carrier for bendroflumethiazide and trichlormethiazide was slightly lower than for MTZ and that it was lower for chlorthalidone than for any of the other thiazides. Again, the profiles seen were similar between the orthologs (TABLE 1).

##### 4.5.1.2. ion dependence.

In two of the additional studies carried out, one by Vázquez et al. (112) and the other by Monroy et al. (108), the characteristics of thiazide binding by flNCC and rtNCC were also tested by determining the ion dependence of Na^+^-Cl^−^ cotransport inhibition at given concentrations of the diuretics. In these experiments, the lowest ion concentrations used in the external medium were 2 and 10 mM for Na^+^ and 2 and 5 mM for Cl^−^. All in all, the authors found inverse correlations between the external concentration of either ion and the affinity of NCC for the thiazide tested.

In the same two studies, the Na^+^ flux of water-injected oocytes was subtracted from the Na^+^ flux of NCC-injected oocytes to correct for background. However, NCC expression could have led this background to change by affecting the activity of other Na^+^ transport pathways. The data as presented are thus difficult to interpret without knowing how much the NCC-independent Na^+^ flux differed between control and test oocytes at the concentrations of inhibitors tested.

The observations made by Vázquez et al. and Monroy et al. on the ion dependence of thiazide binding were again partly inconsistent with those of Tran et al. (41) and Beaumont et al. (34) in the late 1990s. As mentioned above, the earlier studies had shown that the affinity of NCC for different thiazides was negatively correlated with [Cl^−^]_o_ but positively correlated with [Na^+^]_o_. It is in fact through the work of Tran et al. and Beaumont et al. that thiazides and Cl^−^ were said to compete for the Cl^−^ binding site of NCC.

Inhibition of NKCC1 by loop diuretics has also been said to be Na^+^, K^+^, and Cl^−^ dependent. For instance, previous studies by Haas et al. (146) in duck red blood cells and Turner and George (147) in rabbit parotid showed that the dependence of bumetanide inhibition on [Cl^−^]_o_ was biphasic, i.e., that *K*_i(bumetanide)_ decreased as a function of [Cl^−^]_o_ in the lower ranges of [Cl^−^]_o_ and increased in the higher ranges. Turner and George further showed that *K*_i(bumetanide)_ decreased as a function of [Na^+^]_o_ and [K^+^]_o_ regardless of concentration. From these observations, it was concluded that inhibitor and Cl^−^ also competed for a common (translocating or nontranslocating) Cl^−^ binding site in NKCC1.

The question of whether thiazide binding to NCC could be affected through changes in [Na^+^]_i_ or in [Cl^−^]_i_ does not appear to have been addressed in the literature available. In the case of NKCC1, however, transport studies carried out in isolated parotid membrane vesicles by Moore et al. (148) showed that the Cl^−^ binding site of bumetanide (or at least part of it) could be localized in an intracellular domain of NKCC1. That NCC could harbor a conserved site at this location is therefore a possibility.

##### 4.5.1.3. residues involved in thiazide binding.

The approach used to identify thiazide binding sites in NCC was also based on the characterization of rat-flounder chimeras in the *Xenopus laevis* oocyte expression system. It was similar to the approach exploited by Isenring et al. (119, 134–136) to identify loop diuretic binding sites in NKCC1 except for the expression system used. The goal of these studies was to pinpoint the residues/domains that specified the characteristics of drug inhibition in a given CCC species and that could thus be involved in drug binding.

In NKCC1, bumetanide affinity was seen to be specified by residues in TMD2, TMD4, TMD7, TMD11, and TMD12 (119, 134–136). Because Cl^−^ affinity in these studies had been seen to be specified by residues in TMD2, TMD4, and TMD7 more specifically, it was concluded that loop diuretics did not exclusively interact with their target through a Cl^−^ transport site. As for NCC, thiazide affinity was seen to be specified by a single residue in TMD11, that is, a Ser at position 575 in rtNCC and a Cys at the corresponding position in flNCC (110, 111).

On the surface, these differences between NCC and NKCC1 in the localization of drug affinity sites might once again appear discrepant. In particular, loop diuretics and thiazides share similar three-dimensional (3-D) chemical structures, exhibit Cl^−^-dependent binding properties, and act on targets that are very homologous compared with one another. At the same time, and as explained above, the use of a chimera approach to identify residues/domains of interest does come with limitations.

Finally, the mutations found in Gitelman syndrome have also provided little information on the molecular mechanisms through which NCC interacts with thiazides. As already stated, most of these mutations have been found indeed to result in carrier degradation or instability. In addition, most of them have not been tested to determine either their effect on the sensitivity to thiazides or the conformation of the cotransporter.

#### 4.5.2. Mercury.

While flNCC was in the process of being cloned, Wilkinson et al. (149) found that mercury salts could inhibit the Na**^+^**-Cl**^−^** cotransport mechanism in isolated winter flounder bladder. This effect was seen to occur at submicromolar doses of Hg^2+^, to be of rapid onset, and to be entirely reversible. It was explained tentatively by the existence of a binding site for this ion on the cotransporter.

In their study on the side-by-side functional characterization of flNCC and rtNCC, Vázquez et al. (112) made similar observations. They found indeed that in the presence of 50 μM HgCl_2_ on the external side, the transport activities of the fish and rodent orthologs decreased by 60% and 40%, respectively. These results were otherwise in keeping with those of previous studies in which Hg^2+^ was found to inhibit the activity of other CCCs such as NKCC1 (150).

As it stands, the mechanisms that account for the effects of Hg^2+^ ions on NCC and NKCC1 are not completely deciphered. They could be numerous given that these ions are known to form noncovalent bonds with available cysteinyl sulfhydryl (SH^−^) groups (150) and that they could thus affect the activity of almost any proteins including the intermediates through which the function and processing of these transporters are regulated.

In the case of NKCC1, Jacoby et al. (150) have shown that one of the mechanisms at play appeared to involve SH^−^ groups within the residue sequence itself and that these groups were located in the distal portion of TMD11 (near the EL) as well as in the intracellular COOH terminus (Ct). Although, interestingly, flNCC is more sensitive to Hg^2+^ inhibition than rtNCC, it also encloses cysteine residues in TMD11, while the mammalian ortholog does not.

### 4.6. Structure of NCC

#### 4.6.1. Indirect evidence.

##### 4.6.1.1. general structure.

The predicted structural model of NCC has already been discussed in sect. 3.1. As mentioned, it was initially found to consist of an ∼1,000-residue polypeptide that is comprised (from Nt to Ct) of a cytoplasmic domain, a 12-TMD central core, and another cytoplasmic domain. This model was also seen then to be very similar to that predicted for NKCC1 and NKCC2, a finding that came as no surprise given that all members within the Na^+^-coupled CCCs share ∼56% identity and 81% similarity in residue sequence with one another (2, 114–118, 151).

The proposed topological sketch of NCC, i.e., the cellular localization of the main three domains, was partly confirmed later on by uncovering the role of certain residues in the rat or human sequence. In the predicted Nt of rtNCC, for instance, T_53_ and S_71_ were found to act as phosphoregulatory sites (152, 153) and residue stretch 17–22 as an interacting site for a regulatory kinase (154–156). In the predicted TMD of rtNCC, N_404_ was also shown to be N-glycosylated (157) and several domains to be important in specifying ion and thiazide affinities. In the predicted Ct of huNCC, finally, S_811_ was found to act as a phosphorylation site (86, 87) and K_828_ and K_909_ as potential ubiquitination sites (158).

Before cryo-EM-based determinations became available (see below), most of the insight gained into the topological organization of the ion-transporting CCCs still came from the extensive characterization of NKCC1. Two studies were of particular interest in this regard. One of them, which was conducted by Gerelsaikhan et al. (159) through scanning cysteine mutagenesis, allowed confirmation that NKCC1 comprised 12 TMDs flanked by intracellular extremities. The other study, which was conducted by Simard et al. (160) through yeast two-hybrid screens, led to the identification of self-interacting segments that were dispersed throughout the predicted Ct.

##### 4.6.1.2. assembly into homodimers.

In the late 1990s, Moore-Hoon and Turner. (161) were able to demonstrate that NKCC1 was present in biotinylated rat parotid membranes as a homodimeric structure. These investigators found more specifically that treatment of these membranes with a reversible cross-linker called dithiobis(sulfosuccinimidyl propionate) (DTSSP) led NKCC1 to migrate at the 350- rather than 170-kDa landmark on SDS-PAGE gels. Importantly, immunoprecipitation of cross-linked NKCC1 had revealed no other protein by avidin blotting, silver staining, and two-dimensional (2-D) electrophoresis.

During the same period, McKee et al. (162) used a similar approach to show that NCC and NKCC2 in rat kidney membranes were also organized as homodimers. In this other study, treatment of the renal extracts with different types of homobifunctional cross-linkers caused both transporters to form higher-molecular weight structures based on Western blot analyses. Interestingly, NCC and NKCC2 were also detected in rat urine and found to exist in this humor mainly in the form of homocomplexes.

Soon after, de Jong et al. (163) conducted analogous experiments in NCC-expressing oocytes to confirm former observations. In particular, the reversible chemical cross-linker dithiobispropionimidate (DBP) was seen to cause the transporter to migrate at the 300- rather than 140-kDa landmark. Dimerization was confirmed further by showing that a HA-tagged version and a FLAG-tagged version of rtNCC could be coimmunoprecipitated with one another when coexpressed in the same oocyte.

#### 4.6.2. Cryo-EM determinations.

##### 4.6.2.1. general structure.

In 2019, Chew et al. (164) published the first 3-D cryo-EM density map of a CCC family member, i.e., of *Danio rerio* (dr) NKCC1 (drNKCC1). In the following 2–3 years, eight independent studies reported new high-resolution cryo-EM-based structures for all of the ion-transporting CCCs including human NKCC1, KCC1, KCC2, KCC3, KCC4, and NCC (165–172) as well as mouse KCC2 and KCC4 (166, 173). All of the structures resolved were found to exhibit the same overall architecture.

The determinations conducted confirmed several of the past findings. As illustrated in part in FIGURE 2, *B* AND *C*, they showed in essence *1*) that the extremities of the ion-transporting CCCs were located in the cytosol and rich in phosphoacceptor sites, *2*) that the central cores were folded into 12 TMDs, *3*) that the extracellular TMD7-TMD8 loop (for the Na^+^-dependent CCCs) was glycosylated, *4*) that the ion-transporting CCCs were organized in the membrane as homodimers, and *5*) that two of the formerly identified self-interacting domains in the Ct (linker helix α0 and strand β3/helix α3) did consist of dimerization contact points.

The structures published were yet unexpected in several regards. In particular, and as partly illustrated once more in FIGURE 2, *B* AND *C*, they revealed that the TMDs *1*) adopted a Leu-T fold conformation with two sets of inverted repeats in each monomer, *2*) were connected to the Ct of the opposite monomer in a domain swap configuration via EL1, and *3*) were connected with one another via their distal portion (TMD11 and TMD12) to realize a large, outward-facing, horseshoe-shaped cavity. The dimerization interface was also seen to form an inverted V-shaped structure (helix-turn-helix) and the segment just after TMD12 a helix-scissor domain.

Another structural feature of the CCCs was that each of the monomers enclosed a solvent-accessible vestibule delimited by a bundle domain (made up of TMD1, TMD2, TMD6, and TMD7) and a scaffold domain (made up of TMD3, TMD5, TMD8, and TMD10). In the high-order configuration, these vestibules were also found to be relatively far from one another (see FIGURE 2*C*) such that they would each act presumably as an independent translocation pathway within the ensemble.

It is of note that the structures analyzed thus far were almost all in an inward-open state while occluding the ions to which they are bound. The intracellular solvent-accessible cavity formed in this state was found for huNCC to be delimited by polar residues that could serve as an exit pathway for ions between TMD and cytosol (168). As for the extracellular portion of this vestibule, it was seen in the same inward-open state to be gated by a conserved salt bridge between TMD1b (R_158_) and end of TMD3 (E_240_). Above this bridge was also seen a hydrophobic constriction site that could be key in preventing ion back leak from TMD to blood.

Recently, the structures of two CCCs were solved in the outward-open state, that is, of bumetanide-bound huNKCC1 and VU0463271-bound huKCC1 (169, 171). For NKCC1, it was found to be architecturally very similar between the two configurations. One exception was that in the outward-open state the extracellular gate was wider and the TMD1b-TMD3 salt bridge was disrupted through a change in the conformation of TMD10. Another exception was that the intracellular aspect of the vestibule was now constricted and added with a salt bridge (corresponding to residues R_294_-Q_435_-D_510_-K_624_ in huNCC) through a change in the conformation of TMD6b.

##### 4.6.2.2. ion and water binding sites.

Through the characterization of huNCC by cryo-EM (168), it was otherwise possible to identify binding sites for Na^+^, Cl^−^, and water within the solvent-accessible vestibule. These sites were each found to be specific for a given substrate and to be highly conserved among the ion-transporting CCCs. Interestingly, one of the ion binding sites in huNCC had also been found to act as a K^+^ binding site in the K^+^-dependent CCCs (165, 171).

As depicted in part in FIGURE 2*D*, the vestibule was seen more specifically to enclose seven different sites of varied compositions for the binding of the ions and water, i.e., two for Na^+^, two for Cl^−^, and three for water. These sites are called Na_1_, Na_2_, Cl_1_, Cl_2_, W_1_, W_2_, and W_3_ here. In the conformations captured and under the conditions tested, these observations would therefore indicate that the movement of ions by the K^+^-independent CCC is indeed electrically silent.

It is the Na_1_ binding site of NCC that corresponds to the K^+^ binding site (called K1 here) of the K^+^-dependent CCCs. Na^+^ and K^+^ could thus theoretically compete for the same Na1 or K1 binding site in any given CCC, but their access to this site would have to be controlled by an exclusion mechanism to limit K^+^ binding to Na_1_ in NCC and Na^+^ binding to K_1_ in NKCC1. In huNCC, H_234_ in TMD3 could very well be involved in this selection process given that it corresponds to a Tyr in all other CCCs and is <4.0 Å away from the Na_1_/K_1_ binding site (168). However, other residues would likely have to be coinvolved.

As for the W binding sites, they were predicted to play key roles in the translocation of substrates based on density map simulations. In the inward-open state, these roles would be as follows: *1*) to coordinate Cl^−^ binding to Cl_1_ via interaction of water with W_1_ at Y_386_ (TMD7), *2*) to coordinate Cl^−^ binding to Cl_2_ via interaction of water with W_3_ at N_149_ (TMD1b), N_227_ (TMD3), and S_475_ (TMD8), and *3*) to couple Na^+^ and K^+^ translocation through the presence of W_2_ near the Na1 binding site and H_234_ (TMD3).

Another role of the W binding sites could be to convey on NCC the ability of translocating water directly. This possibility is supported by the presence of W_3_ on the cytosolic side of the solvent-accessible vestibule in the inward state. It is also supported by the previous transport studies in which NKCC1 was shown to promote ion-coupled water movement against unfavorable osmotic gradients (127–130). In the cryo-EM studies reported, however, there was no mention of aquaporin-like structural elements that would prevent water from being restricted to the membrane by forming H^+^ bonds with residue side chains.

Finally, structural dynamic analyses provided evidence that ion binding by the CCCs was indeed cooperative. In conjunction with the topology of the interacting sites for ions and water, these findings would suggest at first glance that substrate binding within the solvent-accessible vestibule is indeed ordered rather than random. However, the simulations carried out thus far were insufficiently sensitive or granular to draw any firm conclusions in regard to this possibility.

##### 4.6.2.3. inhibitor binding.

Before the structure of the CCCs came to be known, there were many lines of indirect evidence to suggest that thiazides prevent ions from entering the solvent-accessible vestibule by interacting with NCC on the extracellular side. For cryo-EM studies to provide insight into the structural determinants involved, they would have thus required this transporter to be captured in the outward-open state. As stated above, however, the only report available is based on the characterization of NCC in the opposite configuration.

Lessons can still be learned from the structure of bumetanide-bound NKCC1 and VU0463271-bound KCC1 (169, 171). Indeed, both drugs were found to interact exclusively with the active and outward-open forms of their respective target transporters and, in doing so, to arrest the solvent-accessible vestibule in this configuration. One could thus expect thiazides to exert the same (or similar) structural and functional effects on NCC.

Both bumetanide and VU0463271 were found more specifically to be wedged into a specific subpocket of the translocation pathway and to prevent Cl^−^ from accessing Cl_2_. Interestingly, the TMDs involved were also seen to be the same between NKCC1 and KCC1, i.e., to consist of TMD1b, TMD6a, TMD3, and TMD10 in both transporters. As for the drug-interacting residues, they were found to include a number of the ones that were found to play a role in ion coordination.

##### 4.6.2.4. structural determinants involved in regulation of ncc.

As it stands, very little is known regarding the structural mechanisms through which the CCCs are regulated in response to a change in their phosphorylation state. For the NKCC1 and the KCCs, it has been proposed that these mechanisms involve an allosteric network that is driven by key interactions between the Ct domain of one monomer and the IL1 and/or Nt domains of the opposite monomer (174). It is also of interest that the Ct domains of all CCCs have been recently found by cryo-EM determinations to harbor a bona fide ATP binding site (174).

#### 4.6.3. Structure-function analyses in light of the cryo-EM data.

It would appear that the former characterizations of NKCC and NCC chimeras failed to identify the actual substrate binding residues of the Na^+^-coupled CCCs. However, they were not carried out in expecting the opposite outcome given that such residues were bound to be highly conserved among isoforms and orthologs because of their critical role. The chimeras were exploited instead to identify affinity-modifying TMDs/residues as markers of where the translocation pocket might be located. As it turns out, the structural studies have now shown that the central core of the CCCs forms a tunnellike structure in which most of the TMDs are involved.

The affinity-modifying TMDs/residues uncovered were also predicted to affect the transport cycle by relaying allosterically induced residue-specific conformational states to the binding sites or relaying conformational responses to substrate binding. Their role would then be to modulate various kinetic parameters such as binding on-rates and off-rates, translocation rates, and so on. Future studies aimed at determining the structural repercussions of the affinity-modifying sites could thus shed much light on the molecular dynamics of the solvent-accessible vestibule.

Many of the cryo-EM determinations reported were interpreted in the light of structure-function correlations to confirm the predicted role of certain residues. These analyses yielded limited insight, as a large number of the mutants characterized were only tested for baseline transport activities. Some of the analyses also yielded conflicting results. In the case of NCC, for instance, a H234Y substitution in TMD3 did not cause cation selectively to change as anticipated (168). In fact, this substitution (which had already been linked to Gitelman syndrome) was seen to disrupt bioprocessing of the cotransporter.

### 4.7. Regulation

#### 4.7.1. Regulatory sites.

##### 4.7.1.1. phosphoregulatory sites.

Ion transport by NCC has been shown to be activated through phosphorylation of S/T residues in the Nt of the transporter (175, 176). As shown in FIGURE 2*A*, these residues are now known to include T_53_, T_58_, and S_71_ in both rat and mouse and T_46_, T_55_, and T_60_ in human. They are thus disposed in a cluster that encompasses <30 residues. Of note, T_55_ and T_60_ in human (which correspond to T_53_ and T_58_ in rodents) are conserved among the Na^+^-dependent CCCs, but the other S/T residues within the cluster are not.

Through a study by Tutakhel et al. (86), another phosphoacceptor site of potential relevance was identified in the Ct of NCC. It consists of S811 and is only present in primate-specific NCC1/NCC1B and NCC2/NCC1C (see FIGURE 2*A*). It was ascribed a role in regulation by finding that T_55_, T_60_, and S_811_ in NCC1/NCC1B_WT_ and NCC2/NCC1C_WT_ exhibited greater levels of phosphorylation under hypotonic conditions than did T_55_ and T_60_ in NCC1/NCC1B_S811A_ and in NCC2/NCC1B_S811A_. In this study, however, the three NCC_WT_ variants were found surprisingly to exhibit similar levels of phosphorylation at T_55_ and T_60_ under hypotonic condition.

There are probably many more phosphoacceptor sites of functional importance in NCC. For instance, large-scale phosphoproteomic analyses have led to the identification of >15 Ser, Thr, or Tyr phosphoresidues in the mouse and rat cotransporters (177, 178). In both species, these residues included T_44_ (the equivalent of T_46_ in human) as well as T_53_, T_58_, and S_71_. NCC is also predicted to harbor a number of casein kinase II as well as PKC phosphorylation sites (see https://www.phosphosite.org, using SLC12A3 as keyword) and is directly stimulated by PP1 (see below).

There are several lines of evidence to suggest that NCC is activated by phosphorylation through changes not only in its conformation at the cell surface but in its abundance as well. One of the mechanisms at play has been found to consist of a decrease in carrier endocytosis from the plasma membrane (175, 179). However, it is unclear whether the addition of $PO_{4}^{\mathrm{2-}}$ groups to the Nt per se of NCC is required to sustain this mechanism.

##### 4.7.1.2. nedd4-2 binding site.

Nedd4-2 is a ubiquitin protein ligase that regulates the cell surface abundance and trafficking of many membrane proteins (180). To play this role, however, it must typically bind to specific PY motifs (PPXY or LPXY) in its target proteins and add them with polyubiquitin chains at nearby K residues. It has been claimed that NCC corresponds to one such target and that the residues modified by the ligase (see FIGURE 2*A*) are in Ct, i.e., K_809_ and K_890_ for rtNCC and K_828_ and K_909_ for huNCC (158). However, there are no canonical PY motifs in this CCC.

##### 4.7.1.3. spak and osr1 binding sites.

As shown in FIGURE 2*A*, NCC is also endowed with a SPAK/OSR1 binding motif (the same one for both enzymes) in its Nt ∼30 residues upstream of the phosphoregulatory S/T cluster. This motif consists more specifically of a (S/G/V)RFX(V/I)XX(V/I/T/S)XX residue stretch that is minimally comprised of the RFX(V/I) subsequence (155). It consists more specifically of GRFTISTLLS in human, GRFTISTLLG in rat, and GRFTISTLMG in mouse.

#### 4.7.2. NCC-interacting partners.

##### 4.7.2.1. localization.

In this section of the review, the NCC upstream regulators that are the object of further discussions have all been shown to be expressed in the NCC-expressing cells of the distal nephron. They include SPAK, OSR1, the WNK kinases, CaB39/MO25, SORL1, PP1, PP3, and PP4. The evidence substantiating this claim can be found in the studies that are cited throughout the accompanying text.

##### 4.7.2.2. partners per se.

###### 4.7.2.2.1. SPAK/OSR1.

In vitro studies have confirmed that NCC could be phosphorylated by SPAK and OSR1 at the S/T residue cluster in its Nt (156, 176, 181, 182). Interestingly, all of the ion-transporting CCCs except for the splice variant KCC2B have been shown to harbor at least one RFX(V/I) residue stretch in their Nt and to act as phosphoregulatable substrates for these enzymes (183). As for SPAK/OSR1, they bind to the CCCs through a highly conserved domain in their Ct (184).

**FIGURE 5.**
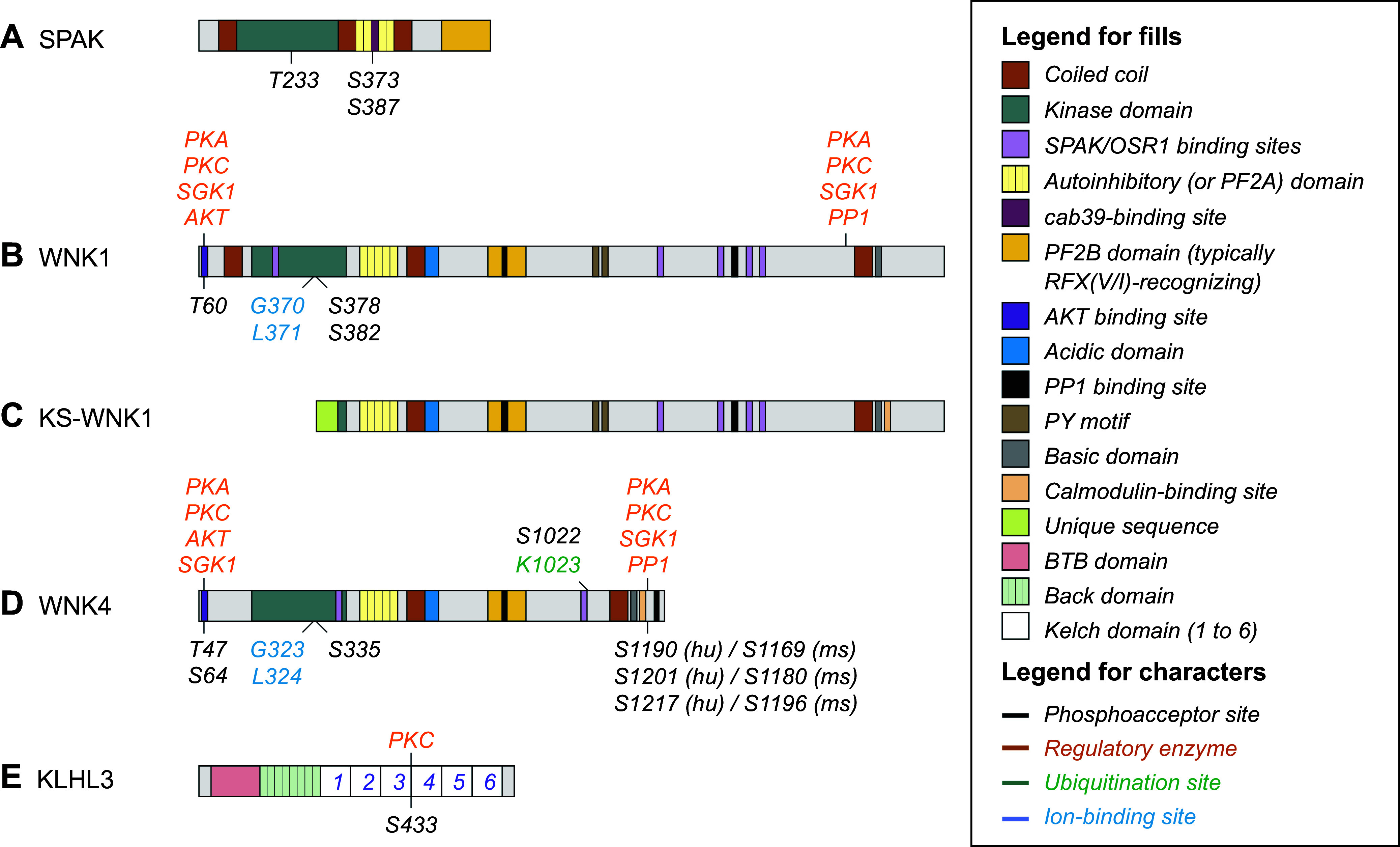
Domains of proteins that are involved in Na^+^-Cl^−^ cotransporter (NCC) regulation. Color code used is indicated at *right*. *A*: SPAK. *B*: WNK1. *C*: kidney-specific (KS)-WNK1. *D*: WNK4. *E*: KLHL3. See glossary for additional abbreviations.

SPAK and OSR1 belong to the germinal center kinase VI subfamily of STE20 kinases (154, 175, 176, 185) and share high levels of homology with one another. As seen in FIGURE 5*A*, they are comprised (from Nt to Ct) of a coiled-coil domain, a T-loop kinase domain with a Thr phosphoacceptor site (T_233_ in SPAK, T_185_ in OSR1), a second coiled-coil domain, an S motif with Ser phosphoacceptor sites (S_373_–S_387_ in SPAK, S_325_–S_339_ in OSR1), a third coiled-coil domain, and an RFX(V/I)-recognizing COOH-terminal domain. The S motif acts as an autoregulatory domain, but the role of its phosphoacceptor sites in this regard is unclear (182, 186).

SPAK- and OSR1-null mouse models have shown that NCC phosphorylation in the DCT is more dependent upon SPAK (187–189) than it is upon OSR1 (190). Along the same line, elevated BP due to upregulation of NCC has been described in mouse models that express high levels of SPAK activity in their DCT (191, 192). One must bear in mind that these observations do not exclude a role for OSR1 in NCC regulation given that they were from characterizations in which the animals tested were not confronted with a range of challenges or pathological conditions.

SPAK/OSR1 cannot phosphorylate NCC unless they are converted into operational enzymes through a series of posttranslational modifications. In particular, they must undergo phosphorylation of their T-loop domain (see FIGURE 5*A*) through the interventions of other enzymes, including most notably the WNK kinases (156, 176, 182, 193–195). They must also interact with Ca^2+^ binding protein 39 (CaB39/MO25) to assemble into stable homodimers (196–198) and with a scaffold protein called protein-related receptor with A-type repeats 1 (SORL1) to be properly sorted at the cell membrane (199).

Aside from SPAK/OSR1, many other enzymes are expected to be involved in direct phosphorylation of NCC. In particular, the phosphoproteomic studies have revealed the presence of several phosphorylated Y residues in the carrier sequence and in silico analyses the presence of consensus sites for a variety of kinases. In addition, functionally relevant PKA, SYK, and AMPK phosphorylation sites have been identified in the other Na^+^-dependent CCCs (200–203).

Finally, it should be mentioned that the CCCs are not the only substrates of SPAK/OSR1 (204–207). For instance, the human β_2_-adrenoreceptor has been recently found to harbor an RFX(V/I) motif that allows its phosphorylation by these enzymes (208). Similarly, many of the human inward-rectifier K^+^ channels have also been found to contain an RFX(V/I) motif in their Ct and two of them (Kir2.1 and Kir2.3) to exhibit heightened cell surface expression upon binding to OSR1 (209, 210).

###### 4.7.2.2.2. Phosphatases.

Although NCC has not been found to harbor strong PP consensus sites, it does appear to be directly regulated by at least two such enzymes, i.e., PP1 and PP4. PP3 has also been found to affect the activity of this transporter but by acting through upstream intermediates. The role of PP1 and PP4 are discussed in this subsection and that of PP3 below in sect. 4.

The evidence that PP1 acts as a direct regulator of NCC comes from various sources. For instance, these two proteins have been found to interact with each other in MDCK cells based on yeast two-hybrid and coimmunoprecipitation studies (211). Along the same line, NCC activity is known to be stimulated by cAMP and this response to increase both I-1 dephosphorylation and carrier phosphorylation (at residue T_53_) in mouse kidney preparations (211–213). Finally, mice inactivated for I-1 were additionally found to exhibit decreased NCC phosphorylation but no change in carrier abundance (211, 214). The topic of NCC regulation by the cAMP/I-1/PP1 cascade is also the subject of further discussion in a sects. 4.7.3.4.2.2.3, 4.8.1.3.1.1, and 4.8.1.3.1.2.5.

As for the involvement of PP4 in NCC regulation, the evidence was obtained in a study where WT and mutant versions of these proteins (rodent NCC_WT_, rodent NCC_T58A_, PP4_WT_, and PP4_dead_) were heterologously coexpressed in *Xenopus laevis* oocytes either alone or in various combinations (215). Na^+^-Cl^−^ cotransport under such conditions was found to be greatly reduced in NCC_WT_/PP4_WT_-expressing eggs but not in NCC_WT_/PP4_dead_- or NCC_T58A_-expressing eggs while NCC abundance at the oocyte surface was the same among these conditions. It was thus concluded that PP4 regulates NCC activity by dephosphorylating the transporter at the cell surface.

###### 4.7.2.2.3. Nedd4-2.

The NCC-Nedd4-2 interaction was revealed through coimmunoprecipitation and ubiquitination studies while heterologously expressed in HEK-293 cells (216). As mentioned above and shown in FIGURE 2*A*, the Ct of this carrier was additionally shown later on to harbor ubiquitination sites but to be devoid of PY motifs (217). It is also of note that Nedd4-2 has the ability to accelerate the turnover of certain WNK1 splice variants and that the same variants are known to regulate SPAK/OSR1 activity (218).

#### 4.7.3. Upstream regulators of SPAK/OSR1: the WNK kinases.

##### 4.7.3.1. generalities.

Members of the WNK kinase family are the main regulators of SPAK/OSR1. They activate both of these substrates by adding them with a $PO_{4}^{\mathrm{2-}}$ group in the T-loop domain. The residues targeted consist of T_233_ in huSPAK and T_185_ in huOSR1 (see FIGURE 5*A*). There is no evidence to indicate that the WNK kinases can associate with NCC directly or with any other CCCs for that matter. As such, they act on the function of these transporters indirectly.

Two members within the WNK kinase family (WNK1 and WNK4) have drawn considerable interest since the early 2000s, as they were found then to be associated with a hereditary disorder called pseudohypoaldosteronism (PHA) type II (or Gordon syndrome) that manifests as a form of hyperkalemic, acidemic, and hypertensive renal tubulopathy resulting from excessive NCC activity along the DCT.

Of importance, many substrates other than SPAK/OSR1 have been found to be regulated by the WNK kinases. Four such substrates are of interest given that they are expressed in the DCT and contribute to the manifestations of PHAII as discussed below. They consist of Nedd4-2, ENaC, TRPV5, and ROMK (219). Given that Nedd4-2 is among the substrates listed, it would thus appear that ligase and kinase are capable of affecting the activity of each other, i.e., the half-life of WNK1 decreases under the effect of Nedd4-2 (as stated just above) and the activity of Nedd4-2 increases under that of WNK1 (218, 220).

##### 4.7.3.2. wnk kinase family.

WNK1 was uncovered by Xu et al. in 2000 (221). Its structure (schematized in FIGURE 5, *B* AND *C*) was found to be atypical for a member of the S/T kinase family given that the ATP binding catalytic lysine was not located in subdomain II as for other kinases but in subdomain I. Xu et al. had demonstrated then that WNK1 was still able to catalyze the phosphorylation of substrates. Soon after, three additional WNK kinase isoforms (called WNK2, WNK3, and WNK4) were identified (222, 223) and found each to be encoded by a unique chromosomal locus (see FIGURE 5*D*, where the structure of WNK4 is also schematized).

As alluded to above, several WNK splice variants were identified early on. One such variant, which is called kidney-specific WNK1 (KS-WNK1) and is DCT specific, has drawn much attention by having been found to regulate both WNK1 and NCC activities (224, 225). As shown in FIGURE 5*C*, it is identical to WNK1 except for lacking most of its Nt (with most of the kinase domain) and for harboring a unique 30-residue header sequence (226). Additional variants have also been found to lack exon(s) 9, 11, 12, and/or 26 (227). Among them, WNK1-E11 is the most abundant one in the kidney but is devoid of a PY motif through the absence of E11 (FIGURE 5*B*) (218, 228).

Soon after KS-WNK1 was identified, many investigators began using the term “long WNK1” (or L-WNK1) to refer to the gene product that was not devoid of its Nt and that was not kidney specific either. This nomenclature is nonetheless confusing given that it is unclear to which variant(s) L-WNK1 refers, i.e., to the one that contains all of the exons or to all of those that do not lack most of the catalytic domain. For clarity, the term “L-WNK1” will not be used in the remainder of this review.

##### 4.7.3.3. structure of the wnks.

As seen in FIGURE 5, *B* AND *D*, the WNK kinases are each comprised of (from Nt to Ct) a T-loop kinase domain with Ser phosphoacceptor sites (S_378_ and S_382_ in huWNK1; S_335_ in huWNK4), an autoinhibitory FXF domain [also known as an RFX(V/I)-recognizing PF2A domain], a negatively charged acidic motif, an RFX(V/I)-recognizing PF2B domain, a basic motif, and a CaM binding site (156, 229, 230). They are comprised in addition of at least two RFX(V/I) SPAK/OSR1 binding sites [1 in their catalytic domain and the other(s) in Ct], of one to several putative coiled-coil domain(s) (the most distal one acting perhaps as WNK binding domain), and of PP1 binding sites.

The WNK kinases are expressed in cells as homodimers by associating with one another via their distal coiled-coil domain. However, they can also form heterodimers that are composed of various isoforms or splice variants including KS-WNK1/WNK1, WNK1/WNK4, WNK3/WNK4, and so on. Within such assemblies, it is believed that the activity of an isoform can be altered by the opposite isoform through dominant-negative/-positive effects or changes in phosphorylation.

##### 4.7.3.4. regulation of ncc by the wnk kinases.

###### 4.7.3.4.1. Models of regulation.

####### 4.7.3.4.1.1. initial model of regulation.

In the early 2000s, PHAII-causing mutations in WNK1 were said to be gain of function. The first one to be identified consisted of a large deletion within intron 1 (222, 231, 232) that led WNK1 to be expressed and phosphorylated at higher levels (222). However, WNK1 was initially shown through functional studies in *Xenopus laevis* oocytes to exert no effect on NCC. It was found instead to decrease the activity of another kinase (WNK4) that acted itself as a negative regulator of NCC (228, 233–237). Activation of WNK1 was thus said then to cause NCC upregulation by inhibiting WNK4.

In contrast to PHAII-causing mutations in WNK1, PHAII-causing mutations in WNK4 were initially presumed to be loss of function, that is, associated with a decrease in enzyme activity (238). The first ones to be identified consisted of missense substitutions in the acidic domain of the enzyme just distal to one of the coiled-coil domains (222). They were thus assumed to hamper WNK4-mediated NCC inactivation.

KS-WNK1 was also believed early on to be involved in the pathogenesis of PHAII. In particular, it was found to repress NCC phosphorylation and activity in vitro by exerting a dominant-negative action on the longer variants (224, 235, 239, 240). Given that the loss of intron 1 was not predicted to alter the structure and inhibitory effect of KS-WNK1, it was said to result in a milder form of PHAII for this reason. However, the repercussions of PHAII-causing mutations in the acidic domain of the short variant were unknown at that time.

**FIGURE 6.**
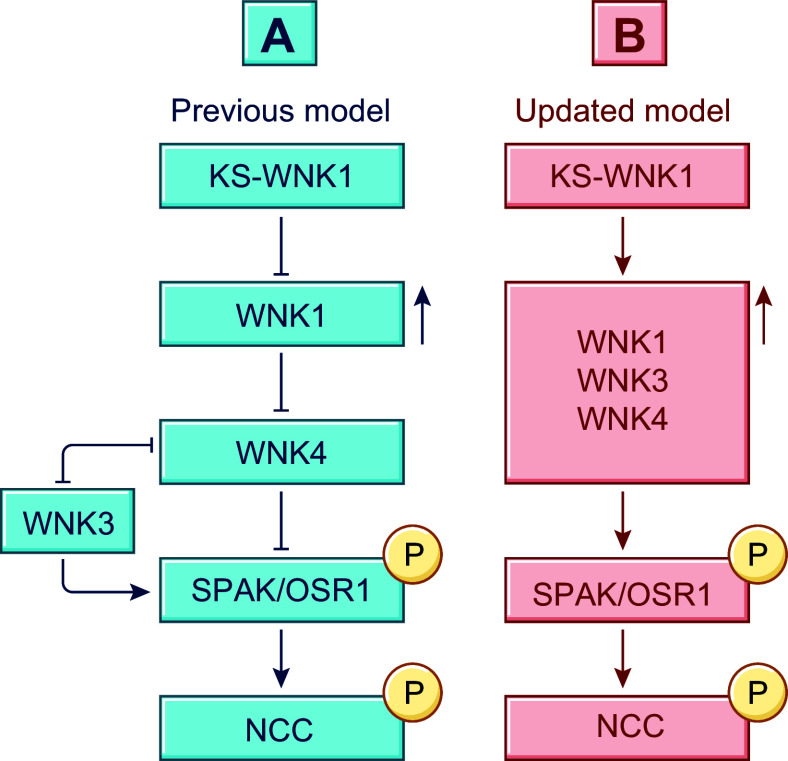
Models of Na^+^-Cl^−^ cotransporter (NCC) regulation. *A*: previous model. *B*: new model. Circled Ps correspond to $PO_{4}^{\mathrm{2-}}$ groups. KS, kidney specific. See glossary for additional abbreviations.

Because of these early (and subsequent) studies on the effects of PHAII-causing mutations and the ability of the WNKs to heterodimerize with one another, a complex model was proposed to describe how NCC is regulated by these enzymes (see FIGURE 6*A*). In this model, KS-WNK1 inhibits WNK1, WNK1 and WNK3 inhibit WNK4, WNK4 inhibits SPAK/OSR1 and/or NCC directly, WNK1 and WNK3 activate SPAK/OSR1, and SPAK/OSR1 activates NCC (233–236). Based on these various interrelationships, gain-of-function mutations in WNK3 would have thus also caused NCC to be upregulated.

The inaugural scheme of NCC regulation by the WNK kinases relied mostly on indirect characterizations in an expression system where the proteins of interest (WT or mutant WNKs) were heterologously overexpressed with NCC. Two additional concerns arose later on. First, the WNK1 cDNA used in many of the experiments carried out was found eventually to harbor an unexpected mutation (219, 228). Second, the effects of WNK expression on CCC activity, expression, or phosphorylation in *Xenopus laevis* oocytes were also found to vary depending on whether SPAK or OSR1 was coexpressed during the studies.

####### 4.7.3.4.1.2. subsequent models of regulation.

After the mid-2000s, WNK1 was shown in a number of studies to exert a stimulatory rather than no effect on NCC (176, 228). In one of these studies, it was even shown to have the ability of acting as such in the absence of WNK4, that is, to increase carrier phosphorylation and cell surface abundance in a WNK4-null model (228). In the same study, in vitro experiments further revealed that WNK1-E11 (the predominant form of WNK1 in the kidney) was able to activate NCC as well and that it did so via the involvement of SPAK.

Based on more recent work, WNK4 was actually found to act as an upregulator of NCC activity and PHAII-causing mutations in this enzyme to be mostly gain of function. It was the characterization of various mouse models that provided the most convincing evidence in this regard. However, the discrepancies that it revealed compared with the results of earlier undertakings are not entirely explicable.

Three of the mouse models that shed light on the role of WNK4 were activated for this enzyme. They consisted of a transgene overexpressing WNK4 (241), an inducible transgene expressing the PHAII-causing WNK4_Q565E_ mutant (242), and a monoallelic knockin expressing the overactive WNK4_D561A/+_ mutant (243). Although all three models were seen to exhibit PHAII and enhanced NCC expression or phosphorylation, the knockin was also seen to exhibit enhanced SPAK/OSR1 phosphorylation.

Five additional mouse models were inactivated for WNK4 (228, 244–247). All were seen to be characterized by a substantial decrease in NCC abundance and activity. One of them had been made hypomorphic for WNK4 by replacement of the WT locus with an enzyme that was devoid of E7, i.e., of the acidic domain. This model was not only found to behave as the other four but also to exhibit decreased SPAK/OSR1 phosphorylation with mild salt wasting under low-salt diet (247).

It should be mentioned that most of the PHAII-causing gene defects in WNK1 were found eventually to also lie in the acidic domain of the enzyme rather than intron 1 and to consist of charge-changing single-residue substitutions (248). The same is now known to hold true in regard to the PHAII-causing gene defects in WNK4. As discussed below, it is now also known that these residue substitutions prevent the acidic domain from binding to a CUL3-interacting adaptor called KLHL3 and from sustaining ubiquitination of the WNK kinases (241, 248–250). That KLHL3 and CUL3 would play such a role is suggested further by the recent identification of PHAII-associated mutations in their encoding genes.

By showing that most if not all of the PHAII-causing mutations in WNK1 and WNK4 cause NCC to be activated indirectly by increasing SPAK/OSR1 phosphorylation, the more recent studies do not fully support the inaugural model of NCC regulation (see FIGURE 6*A*). A newer model is presented in FIGURE 6*B* but will likely require much adjusting in the future given that there are many unknowns pertaining in particular to the regulation, activity, and role of the WNK isoforms or splice variants in the setting of high-order structures.

In the DCT, the WNK-SPAK/OSRI-NCC pathway was ultimately found to play key homeostatic roles during adaptive responses to certain challenges such as dietary K^+^ restriction, K^+^ loading, change in [Cl^−^]_i_, and so on. However, the involvement of WNK4 and KS-WNK1 in NCC regulation under such circumstances was found to predominate over that of the other WNK kinases.

The complexity of the WNK kinase signaling network took a step up when a number of research groups found in the 2010s that WNK4 and SPAK/OSR1 could coalesce into large puncta along the DCT during the adaptive responses (187, 251–255). These puncta, which are now called WNK bodies, correspond to dynamic membraneless foci where KS-WNK1 is attached to the ribosomal protein L22 via its unique Nt domain and where it recruits the other enzymes. The WNK bodies are thus considered to be KS-WNK1-dependent units.

This complexity of the WNK kinase signaling network is illustrated even further by recent studies in which the effect of mutations in the acidic domain of KS-WNK1 was examined. It was found notably that these mutations led the shorter variant to be expressed at higher levels than WNK1 in the DCT and to still endow both the long and short variants with gain-of-function activity, i.e., with the ability of enhancing WNK4 phosphorylation (248).

###### 4.7.3.4.2. Regulation of the WNK.

####### 4.7.3.4.2.1. context.

**FIGURE 7.**
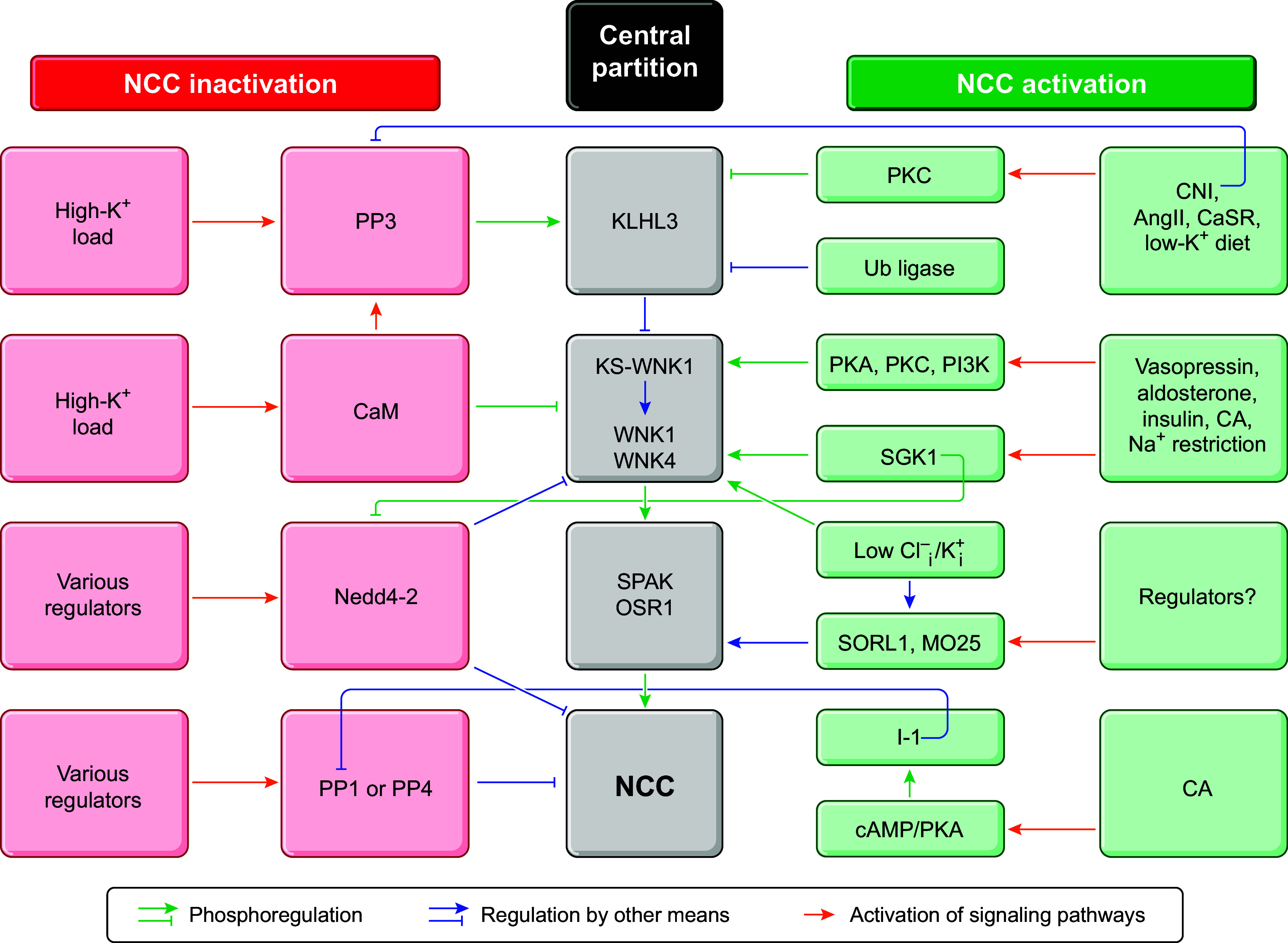
Signaling cascades involved in Na^+^-Cl^−^ cotransporter (NCC) regulation. Color code used is indicated at *bottom*. CA, catecholamines; CNI, calcineurin inhibitor; Ub, ubiquitin. See glossary for additional abbreviations.

In sect. 4.7.3.1.2.2, the various mechanisms that are involved in WNK regulation are the subject of a brief description with the aid of a recapitulative cartoon that is shown in FIGURE 7. This undertaking could be seen as beyond the scope of a review on the molecular characteristics of Na^+^-Cl^−^ cotransport. However, it appears justified in our eyes as many of the NCC-dependent adaptive responses are dictated by the factors that regulate WNK kinase activity and abundance. Among the upstream regulators that are discussed, most have also been identified as residents of both DCT1 and DCT2.

####### 4.7.3.4.2.2. regulators per se.

####### 4.7.3.4.2.2.1. ions and osmotic pressure.

Through the presence of certain residues in their catalytic domain, the WNK kinases are affected by changes in the intracellular concentration of ions such as Cl^−^ and Mg^2+^ (256–258) and are able to be at the center of coordinated homeostatic responses because of this specific property (252, 256, 259). They are more specifically prevented from undergoing autophosphorylation and becoming catalytically active when they bind to either ion directly. Their absence of such residues in KS-WNK1 would thus render this isoform ion insensitive.

For Cl^−^, the residues at work (see FIGURE 5*B*) have been found to include G_370_ and L_371_ in huWNK1 and G_323_ and L_324_ in huWNK4 (256, 258). Terker et al. (260) have also shown that the concentration of Cl^−^ required to inhibit WNK1 or WNK3 was higher than for WNK4 but still lay within a physiological range. This finding would suggest that WNK4 has the ability of regulating SPAK/OSR1 without the intervention of WNK1. The same finding could also explain why WNK4 appears to play a preponderant role in NCC regulation among the isoenzymes.

Another study by Sun et al. (261) also showed that expression of SORL1 was required for Cl^−^ sensing by the WNK kinases to be effective and that it could thus act upon these enzymes in addition to its effect on SPAK/OSR1 (262) (see FIGURE 7). The mechanism at play was not deciphered but could involve cooperative interactions between Cl^−^ and the scaffold protein itself. Another possibility is that the WNK kinases could simply exhibit greater Cl^−^ sensitivity when they interact with SORL1-bound SPAK/OSR1.

The WNKs have been found to be osmosensitive as well (186, 258, 263, 264). They were shown in particular to undergo autophosphorylation (but at residue S_382_ in huWNK1) under conditions of increased osmotic pressure (186) but only in the absence of Cl^−^ (263). Structural studies have additionally shown that the mechanisms at play include a decrease in the hydration and overall cavity sizes of the enzyme (258, 263). The localization of the osmotic-sensitive site in WNK1 is illustrated in FIGURE 5*B*.

####### 4.7.3.4.2.2.2. sgk1.

SGK1 is a widely distributed enzyme and one of the primary mineralocorticoid-induced effectors in the distal nephron (265). It has been found to increase NCC abundance at the cell surface of DCT2 through phosphoactivation of the WNK kinases (218, 237, 266, 267) (see FIGURE 7) at conserved residues in the COOH-terminal domain (266). In msWNK4 these residues would include S_1169_, S_1180_, and S_1196_, and in huWNK4 they would include (by alignment) S_1190_, S_1201_, and S_1217_ (FIGURE 5*D*). Regulation of NCC by SGK1 also appears to implicate Nedd4-2 as seen below (216).

Previous studies have shown that WNK1 could act as an upstream activator of SGK1 (220). However, these findings do not appear to have been confirmed in subsequent publications. Additionally, they were from experiments in which all of the WNK1 variants assayed lacked exons 11 and 12. One could perhaps imagine that WNK1 and SGK1 are both able to act as the substrate of each other but that the enzyme acted upon varies as a function of conformational imperatives based on the presence or absence of E11/E12.

####### 4.7.3.4.2.2.3. pkc/pka.

The WNK kinases are also known to be acted upon by PKA and PKC (268–271) (see FIGURE 7). The residues targeted by these other protein kinases and the consequence of their phosphorylation on WNK activity appear to be the same as for SGK1 (see FIGURE 5, *B* AND *D*). PKA signaling in the DCT can thus lead to NCC phosphoactivation through two different pathways, i.e., through phosphorylation of the WNK kinase and (as explained above) through dephosphorylation of PP1 via phosphorylation of I-1 (see FIGURE 7 once again).

####### 4.7.3.4.2.2.4. cam.

A CaM binding domain is present in the COOH-terminal extremity of the WNK kinases (237, 272, 273). As depicted in FIGURE 5*B*, it is localized in huWNK4 between the last coiled-coil domain (residues 1175 to 1194 from E17) and a PP1-sensitive phosphoregulatory domain (see below). It also encompasses the most proximal PKA/PKC/SGK1-sensitive phosphoregulatory sites in this isoenzyme (S_1190_) but lies just upstream of the more distal ones.

On the basis of experimental evidence and the observation that rare mutations in the CaM binding domain have been associated with PHAII (237, 272, 273), it would appear that the purpose of this domain in the WNK kinases is to limit access of $PO_{4}^{\mathrm{2-}}$ groups to the PKA/PKC/SGK1-sensitive phosphoregulatory sites (see FIGURE 5 and FIGURE 7). When disrupted through specific mutations, the CaM binding domain would be thus be relieved of its negative regulatory role and the WNK kinases undergo irrepressible phosphorylation of their COOH-terminal end.

####### 4.7.3.4.2.2.5. pp1.

Not only has PP1 been found to inactivate NCC through direct dephosphorylation of residue T_53_ in the NH_2_-terminal domain of the rat and mouse sequences (211, 212, 214), it has also been shown to do so indirectly by dephosphorylating the distal end of the WNK kinases (274). In the case of WNK4 more specifically, two PP1 binding sites have been found to sustain this reaction. One is localized just after the CaM binding site and the other just after the acidic domain (see FIGURE 5*D*). As for the residues targeted in the isoenzyme, they would include S_1190_, S_1201_, and S_1217_ based on the human sequence (275, 276).

####### 4.7.3.4.2.2.6. klhl3-cul3.

**FIGURE 8.**
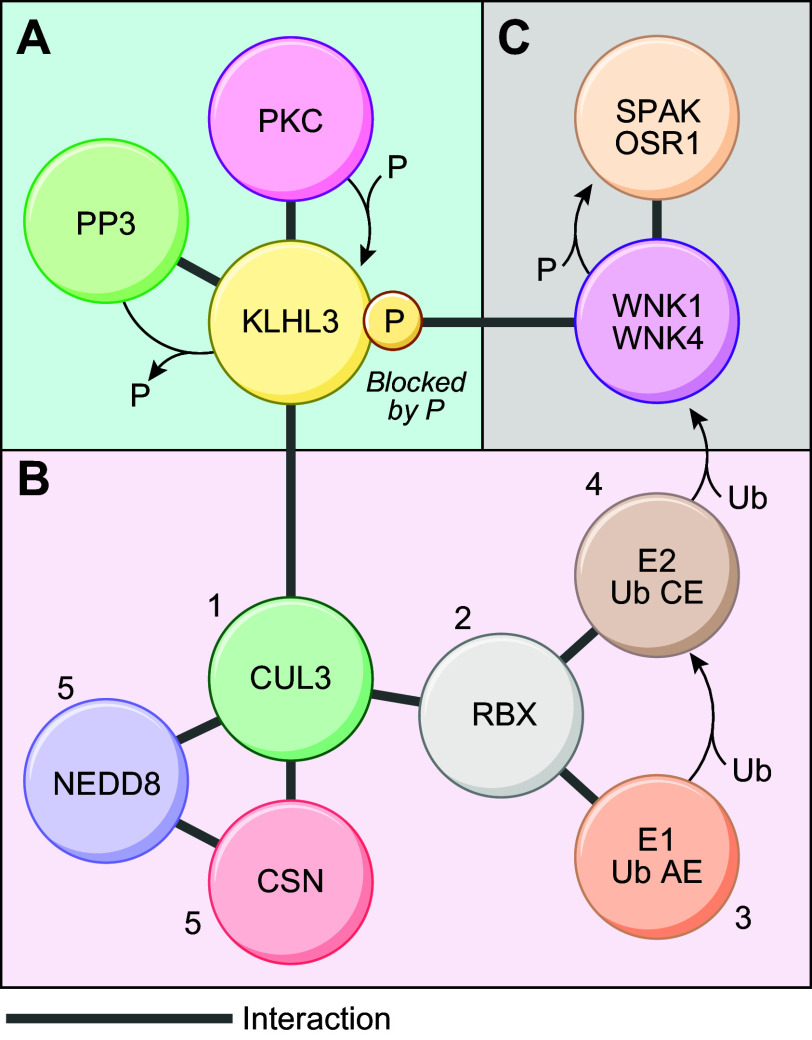
Regulation of the Na^+^-Cl^−^ cotransporter (NCC) by KLHL3 and CUL3. *A*: regulation of KLHL3 by PKC and PP3. KLHL3 is allowed to interact with WNK1/WNK4 when it is dephosphorylated by PP3 but not when it is phosphorylated by PKC (277, 278). *B*: regulation of WNK1/WNK4 by the E3 ubiquitin ligase multimeric complex. E3 ligase is comprised more specifically of *1*) CUL3 that binds to the Bric-a-brac, tramtrack, broad-complex domain (BTB domain) of KLHL3, *2*) ring box protein (RBX) that places KLHL3-bound WNK1/WNK4 close to E2 ubiquitin (Ub) conjugating enzyme (CE) by interacting with both CUL3 and E2 Ub CE, *3*) E1 Ub activating enzyme (AE) that prompts E2 Ub CE to be active, *4*) E2 Ub CE that sustains ubiquitination of Lys residues within physically recruited WNK1/WNK4, and *5*) NEDD8 and COP9 signalosome (CSN) that both allow CUL3 to be active (249, 254, 268, 279,280). When WNK1/WNK4 are thus allowed to interact with KLHL3, they undergo ubiquitination and subsequent degradation by the proteasome. *C*: regulation of SPAK/OSR1 (and NCC as a result) by WNK1/WNK4. P, phosphorylation. See glossary for additional abbreviations.

As already alluded to, KLHL3 not only can associate with the acidic domain of the WNK kinases, it also acts as an adaptor protein for a multimeric complex that is comprised in particular of CUL3 and of an endoplasmic reticulum-based ligase called E2 ubiquitin-conjugating enzyme (see FIGURE 8, *A–C*) (279). Through these interactions, both KLHL3 and CUL3 within the complex have been found to promote ubiquitination of the WNK kinases (at K_1023_ in the case of huWNK4) and cause them to undergo degradation by the proteasome (249, 254, 268, 281) (FIGURE 5 and FIGURE 8). Their role in the DCT is thus to downregulate WNK kinase abundance.

HuKLHL3 harbors a phosphoregulatory Ser residue at position 433 in one of its so-called Kelch domains of the protein (FIGURE 5*E*). When it is added with a $PO_{4}^{\mathrm{2-}}$ group by PKC at this residue site, it is essentially inactive as an adaptor protein, i.e., unable to interact with the WNK kinases, and when it is removed of this moiety by PP3 (i.e., by calcineurin), it can then allow its targets to integrate the CUL3-E2 ubiquitin-conjugating enzyme complex (277, 278) (see again FIGURE 8*A*). KLHL3 itself can also be ubiquitinated for degradation by the proteasome (249).

As alluded above and rediscussed in sect. 4.9 at greater length, loss-of-function variants in KLHL3 have been linked to PHAII by haploinsufficiency (241, 249, 250, 282). They cause their effect by preventing the WNK-KLHL3 or KLHL3-CUL3 interaction from occurring and are thus associated with increased WNK expression in the DCT (281, 283, 284). Deletion of exon 9 in CUL3 has also been linked to PHAII but by causing this protein to become less abundant or interactive (251, 285–288).

Calcineurin (PP3) inhibitors such as cyclosporine and tacrolimus have been shown to cause renal Na^+^ and K^+^ retention by activating NCC (289, 290). It was an interesting study by Ishizawa et al. (291) that shed light on the mechanisms involved. It revealed, for instance, that KLHL3 was a PP3 target in HEK-293 cells and that its dephosphorylation by the enzyme was amplified in a high-K^+^ medium (FIGURE 7) (291). The same study also revealed that mice fed with tacrolimus exhibited increased KLHL3, WNK1, WNK4, SPAK, and NCC phosphorylation activity.

####### 4.7.3.4.2.2.7. nedd4-2.

As mentioned, Nedd4-2 accelerates the turnover of WNK1 through ubiquitination of the kinase (218). For this change to occur, however, the splice variant targeted must include E11 or E12, in that the other exons are devoid of PY motifs (218). As E11/E12-containing variants are robustly expressed in the DCT, these observations imply that Nedd4-2 should decrease NCC activity in vivo (FIGURE 7). SGK1 has also been found to phosphorylate Nedd4-2 directly (218, 226, 227, 292) and TNFα to reduce Nedd4-2 expression (293) such that they should both attenuate degradation of WNK1 + E11/12 by the ligase. Otherwise, WNK4 is devoid of PY motifs and does not appear to be regulated by Nedd4-2 (254, 294).

####### 4.7.3.4.2.2.8. pi3k-akt.

There is now strong evidence to suggest that AKT corresponds to an additional intermediate that can upregulate the activity of WNK1 in the DCT (267, 295–298). It has been found to do so by two different mechanisms, that is, by phosphorylating T_60_ within a precise recognition motif (residues 53 to 64 in the human sequence) into the Nt of the substrate enzyme (FIGURE 5, *B* AND *D*) (267, 297–299) and by preventing WNK degradation through phosphorylation of KLHL3 at residue S_433_ (300). AKT has been found to act more specifically on WNK1 and KLHL3 in response to insulin or vasopressin and to account for the activating effect of these hormones on NCC activity.

### 4.8. Physiological Roles of NCC

#### 4.8.1. DCT.

##### 4.8.1.1. distribution of other ion transport systems and of regulatory partners.

As already stated on several occasions, NCC affects the activity of many ion transport systems and regulatory intermediates throughout and beyond the DCT by altering the electrochemical gradient and intracellular concentration of Na^+^ and Cl^−^. However, the proteins impacted are expressed differentially along the renal epithelium where they reside. As such, they cause each nephron subsegment between DCT1 and the cortical collecting duct (CCD) to also react differentially in the face of altered NCC activity. At this stage of the review, it would thus appear appropriate to provide an illustration of how certain of the ion transport systems are affected by the Na^+^ and Cl^−^ cotransport mechanism and how an enzyme called 11β-HSD2 is partitioned between the macula densa and CCD (4, 301–308).

**FIGURE 9.**
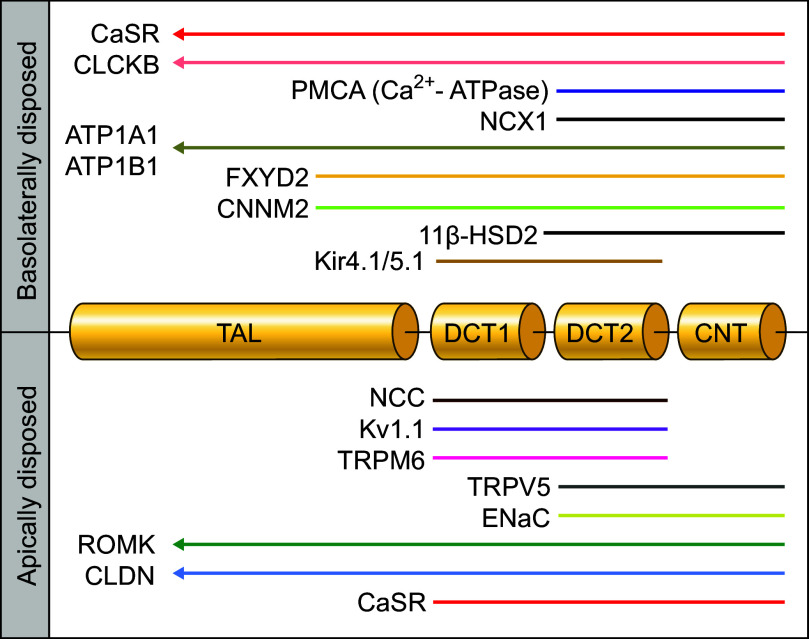
Distribution of ion transport systems and membrane receptors in the distal convoluted tubule (DCT)1, DCT2, and connecting tubule (CNT) nephron subsegments. Expression sites are indicated by horizontal lines for each of the proteins listed. They are based on multiple studies but mostly in rodents (see references in text). Note that several of these proteins have been found to be present outside of the segments encompassed by the horizontal lines but at much lower levels. TAL, thick ascending loop. See glossary for additional abbreviations.

FIGURE 9 is used to this end. It is seen that NCC is present in DCT1/DCT2 and that the same holds true for TRPM6, CNMM2, Kir4.1, Kir4.5, and Kv1.1. Importantly, 11β-HSD2 is predominantly expressed in DCT2/CNT such that the Na^+^ and Cl^−^ cotransport mechanism in DCT1 is not highly sensitive to mineralocorticoids (see sect. 4.8.1.3). As for the Ca^2+^-dependent transport systems, they are seen to be localized in DCT2/CNT, i.e., slightly further down compared with the Mg^2+^-dependent transport systems and CNNM2. One should not lose sight of the fact that the localization data depicted are mostly from mouse or rat studies and that they do not necessarily concord in every respect among reports (4, 301–308).

##### 4.8.1.2. transport roles.

###### 4.8.1.2.1. Primary transport role.

**FIGURE 10.**
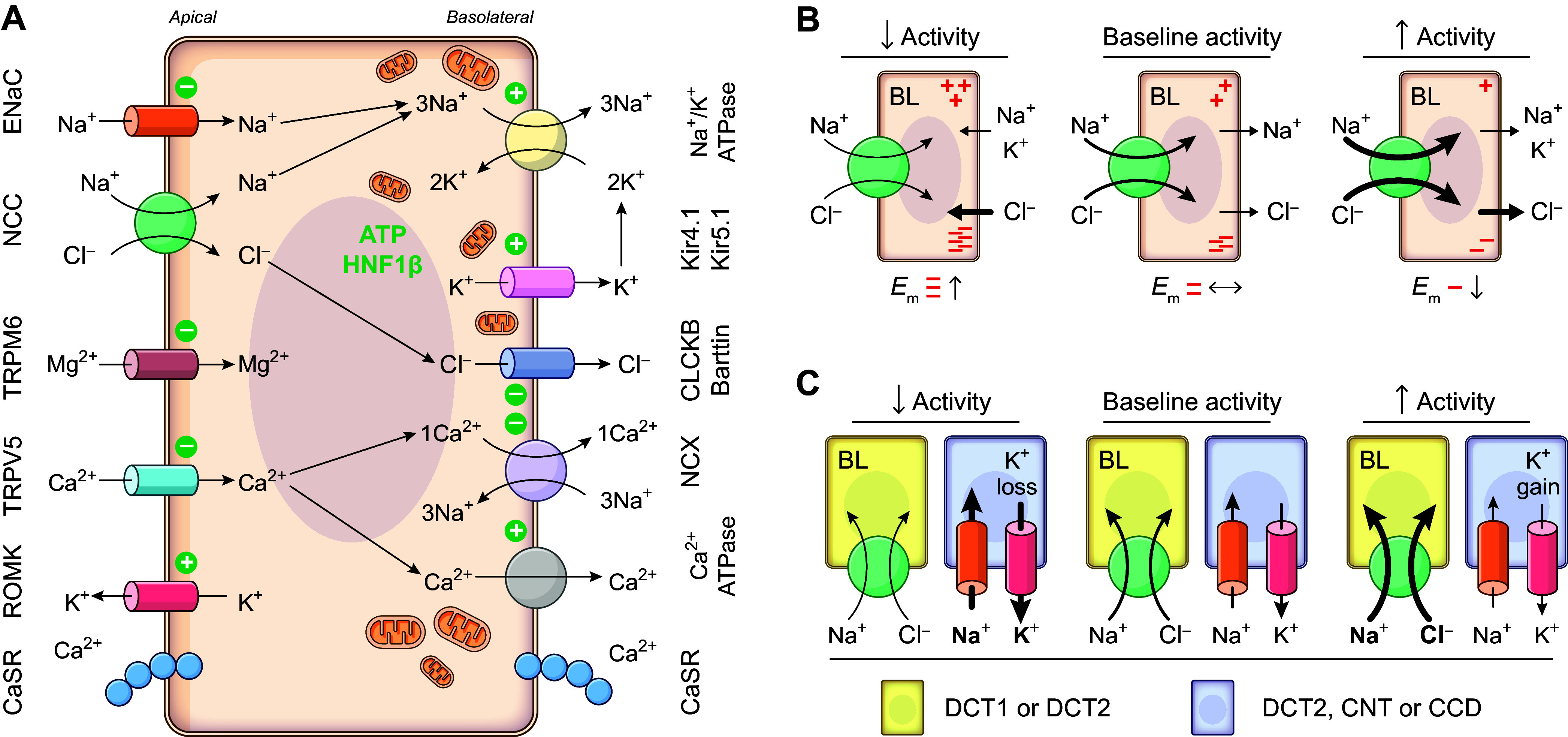
Ion transport by distal convoluted tubule (DCT) cells. *A*: ion transport systems. When the transport systems shown are activated by a decrease in membrane potential (*E*_m_), they are accompanied by + signs in green and when they are activated by an increase in *E*_m_, they are accompanied by – signs also in green. *B*: effect of changes in Na^+^-Cl^−^ cotransport on *E*_m_. *C*: effect of changes in Na^+^-Cl^−^ cotransport on electrogenic K^+^ secretion. BL, basolateral membrane; CCD, cortical collecting duct; CNT, connecting tubule. See glossary for additional abbreviations.

Whereas the apical transfer of the ultrafiltered Na^+^ load in DCT1 and DCT2 is ensured by NCC and to a smaller extent by NHE2, it is also sustained in DCT2 by ENaC and is thus partly electrogenic along these nephron subsegments (4). As for the basolateral transfer of Na^+^ from both DCT1 and DCT2 into the circulation, it is mainly achieved by the Na^+^-K^+^-ATPase pump (α1-β1-FXYD2) (301) and accessorily by some of the NHE isoforms (309–312). The mechanisms of Na^+^ reabsorption in NCC-expressing renal cells are illustrated in FIGURE 9 and FIGURE 10*A*.

In contrast to Na^+^, Cl^−^ depends almost entirely on the cotransport mechanism to enter DCT cells through the apical side and on a Cl^−^ channel called CLCKB to leave them through the basolateral side (see FIGURE 9 and FIGURE 10*A* once more) (301). However, the channel involved in this process must heterodimerize with an accessory subunit called Barttin (BSND) to act as a functional ion transport system in the distal nephron (313).

NCC is also a determinant of *E*_m_ in DCT cells. The mechanisms involved (314–317) are illustrated in FIGURE 10*B*. When NCC becomes less active, the ensuing electroneutral reduction in [Na^+^]_i_ and [Cl^−^]_i_ leads to a net electrogenic increase in the inward movement of these ions and of K^+^ but an increase that is greater for Cl^−^ than for the cations, such that DCT cells become more hyperpolarized (↑*E*_m_). When, contrariwise, NCC becomes more active, the ensuing electroneutral rise in [Na^+^]_i_ and [Cl^−^]_i_ leads to a net electrogenic decrease in the inward movement of these ions and of K^+^ but a decrease that is greater for Cl^−^ than for the cations, such that DCT cells become less hyperpolarized (↓*E*_m_).

###### 4.8.1.2.2. Secondary transport roles.

####### 4.8.1.2.2.1. secondary changes in electrogenic k^+^ transport.

Although NCC does not harbor a K^+^ translocation site (at least not a strong one), it affects the transport of this ion indirectly by impacting on the quantity of Na^+^ that can reach ENaC in ROMK-expressing cells beyond DCT1 (212, 318). FIGURE 10*C* shows how K^+^ secretion varies as a function of Na^+^-Cl^−^ cotransport. It is seen that when the activity of NCC increases along DCT1/DCT2, the ensuing decrease in distal Na^+^ delivery along DCT2 and CNT causes K^+^ secretion to decline but that when this activity decreases, the ensuing increase in distal Na^+^ delivery along DCT2 and CNT causes K^+^ secretion to rise. As discussed below, the activity of NCC can also be affected by its very own impact on K^+^ homeostasis.

####### 4.8.1.2.2.2. secondary changes in ca^2+^ and mg^2+^ transport.

Ca^2+^ reabsorption in the late DCT (that is, in DCT2 and CNT) has been found to vary inversely with Na^+^-Cl^−^ cotransport activity in the earlier DCT (that is, in DCT1 and DCT2). For instance, it has been shown to increase in Gitelman syndrome or through NCC inhibition by thiazides (314, 319) and to decrease in PHAII as well as in hypertensive Mongolian families who would be presumably affected by gain-of-function polymorphisms in NCC (320–322).

Mg^2+^ reabsorption by the DCT1 and DCT2 subsegments has also been found to depend upon the activity of NCC. By way of illustration, it has been shown to decrease when Na^+^-Cl^−^ cotransport is impaired through loss-of-function mutations in the cotransporter itself or in WNK1 (323, 324). However, it does not appear to increase when Na^+^-Cl^−^ cotransport is accentuated through gain-of-function mutations in WNK1 and WNK4 (325, 326), at least not if one relies on serum [Mg^2+^] levels.

It is unclear why the renal Mg^2+^ handling defect of PHAII would not mirror that of Gitelman syndrome or a decrease in WNK1 activity. At the same time, it could be that Mg^2+^ reabsorption by the DCT does increase in PHAII (327, 328) but is blunted in other nephron segments or the gastrointestinal tract through compensatory mechanisms. This scenario could then result in normal urinary and serum [Mg^2+^] even if there is retention of the cation by one of the nephron segments. One must remember that in the absence of renal failure or a primary tubular defect in Mg^2+^ handling, hypermagnesemia is extremely rare and far less common than hypomagnesemia (329).

The mechanisms that account for the dependence of Ca^2+^ and Mg^2+^ transport on NCC are still unclear. They obviously involve a number of the transport systems, binding proteins, and sensors that allow these ions to be reabsorbed by the DCT or nearby nephron segments (see FIGURE 10*A*). For Ca^2+^, the candidates would thus include TRPV5 on the apical side, CALB1 in the cytoplasm, and NCX1 as well as PMCA on the basolateral side. For Mg^2+^, they would include TRPM6 on the apical side, MgBP in the cytoplasm, and CNMM2 as well as Mg^2+^ transporters of unknown molecular identity on the basolateral side (92, 330, 331).

One of the mechanisms proposed for this dependence is that NCC affects the activity of Ca^2+^ transport systems by altering the electrochemical phenotype of DCT2 where NCC is also expressed (314, 319, 332, 333). Under this scenario, and as illustrated in FIGURE 10, *A* AND *B*, a decrease in Na^+^-Cl^−^ cotransport would stimulate the electrogenic entry of Ca^2+^ via TRPV5 by increasing *E*_m_ and stimulate Ca^2+^ exit via NCX1 by the same mechanism and also by decreasing [Na^+^]_i_. The problem with this electrochemical scheme is that Ca^2+^ transport by PCMA in DCT2 should be then compromised and Mg^2+^ entry by TRPM6 in DCT1 and DCT2 enhanced.

Another mechanism could be that the NCC-dependent changes in electrochemical phenotype affect ion- or charge-sensitive signaling intermediates that regulate Ca^2+^ and Mg^2+^ transport in the DCT. Two candidates in this regard could include WNK4 and the CaSR, as both have been shown to exhibit Cl^−^- or ionic strength-dependent sensitive activities (256–258, 260, 264, 334). Evidence for their involvement is that a decrease in NCC activity has been shown to be associated with lower expression levels of TRPM6 and higher expression levels of TRPV5 and NCX1 (331, 332, 335, 336).

A third possibility could be related to the structural changes that occur along the DCT1, DCT2, and CNT subsegments when NCC activity is reduced (337, 338). For instance, chronic administration of thiazides has been shown to induce apoptosis of DCT1 and DCT2 cells (337), where most of the Mg^2+^ load that has passed the macula densa is reabsorbed. A lower number of active DCT cells would then lead to a hypermagnesuric phenotype. Conversely, chronic inhibition of NCC has been shown to induce hypertrophy/hyperplasia of CNT cells (316), where most of the Ca^2+^ load that has entered the DCT is reabsorbed. A higher number of active CNT cells would then lead to a hypocalciuric phenotype.

##### 4.8.1.3. homeostatic roles.

###### 4.8.1.3.1. Responses of NCC to various factors.

####### 4.8.1.3.1.1. premise.

In the DCT, many factors or cues have been found to affect Na^+^-Cl^−^ cotransport and to do so by activating or inhibiting the PKA-I-1-PP1 and KLHL3-WNK-SPAK/OSR1 signaling cascades. Their effects are detailed in the following subsections along with the mechanisms at play. The recapitulative diagram of FIGURE 7 can be consulted upon reading any of these subsections to facilitate integration and keep focus on the larger picture. Note that although some of the effects and mechanisms described have been established from in vitro studies, all of the intermediates required for NCC to be affected by the factors and cues discussed below are known to be expressed in the DCT.

The narrative proposed should also serve as a useful prelude to understand why the purpose of Na^+^-Cl^−^ cotransport in the DCT goes beyond the mere transfer of two ions from lumen to blood and how this purpose is fulfilled in the face of various cues or homeostatic derangements. If, for instance, NCC plays an important role in K^+^ homeostasis, it should then react to a low-K^+^ diet by promoting renal K^+^ retention.

####### 4.8.1.3.1.2. factors per se.

####### 4.8.1.3.1.2.1. aldosterone and dietary nacl restriction.

NCC activity has been shown to increase in response to acute aldosterone infusion or dietary Na^+^ restriction (46, 101, 339, 340). In rat DCT, this response can be blunted by spironolactone such that it is mediated via the mineralocorticoid receptor in DCT2 (280, 341). The intermediates involved have been found to include SGK1, Nedd4-2, WNK4, and SPAK (216, 237, 266, 342–352). As argued later, a decrease in serum [K^+^] resulting from the activation of ENaC in response to aldosterone could also account for the effect of aldosterone on NCC (344, 353–355).

####### 4.8.1.3.1.2.2. angiotensin ii and volume depletion.

NCC abundance in the DCT has been shown to increase in response to acute angiotensin II (Ang II) administration (244, 356–360) and to do so independently of aldosterone (361, 362). The cascade at play appears to involve several intermediates including the Ang II receptor, PKC, PKA, KLHL3, WNK4, and SPAK. In this pathway, PKC prevents WNK4 degradation by phosphorylating KLHL3 (278) and PKC/PKA lead to autophosphorylation of WNK4 by phosphorylating S_64_ and S_1217_ in the enzyme targeted (244).

####### 4.8.1.3.1.2.3. vasopressin.

The administration of vasopressin exerts the same action on NCC as the administration of aldosterone and Ang II (152, 363, 364). When it is acute, the intermediates at work include the vasopressin receptor 2, adenylate cyclase 6, the WNK kinases, and SPAK (365–368). When, by contrast, the administration of vasopressin is chronic, the intermediates at work appear to include CaMK and PI3K among other players (368, 369).

####### 4.8.1.3.1.2.4. insulin.

Insulin is another NCC-activating hormone (295, 296, 370). For instance, animal models of hyperinsulinemia have been found to exhibit higher levels of NCC expression, phosphorylation, and/or natriuresis in response to thiazides (371–374). In two such models, these changes were found to result from the activation of WNK4 by PI3K (295, 296, 298, 375). A decrease in serum [K^+^] resulting from the cellular buffering of this ion in response to insulin could also account for the effect of insulin on NCC.

####### 4.8.1.3.1.2.5. catecholamines.

Norepinephrine has been shown, like insulin, to stimulate NCC and to enhance the effect of thiazides on urinary Na^+^ excretion (376–378). In mice, this response is not mediated by an increase in Ang II but by direct stimulation of the β-adrenergic receptors that leads to phosphorylation of OSR1 by the WNK kinases and of I-1 by PKA (211). A recent study has shown that norepinephrine also caused the *E*_m_ of DCT cells to increase by stimulating basolateral Kir4.1/Kir5.1 channel activity (see FIGURE 7 and FIGURE 9) and that it did not affect NCC if the voltage was clamped (376).

####### 4.8.1.3.1.2.6. casr.

In DCT cells, the CaSR is present on both the apical and basolateral membranes (379, 380). On the basolateral side, it could potentially lead to reduced Na^+^-Cl^−^ cotransport upon stimulation by sponging Kir4.1 (and decreasing *E*_m_ as a result) or by recruiting PP3 (303, 381). On the apical side, it leads to enhanced Na^+^-Cl^−^ cotransport, as suggested by the observation that increased luminal Ca^2+^ or glucose delivery to the DCT (through furosemide and SGLT2 inhibitors, respectively) causes NCC to undergo phosphorylation via activation of the KLHL3-WNK4-SPAK pathway by PKC (303, 328, 382).

It is of note that when cinacalcet is given to mouse or human systemically, it also activates NCC (328, 382) even if it is not excreted in urine (383, 384). However, it presumably still leads to stimulation of the apical CaSR of DCT cells by increasing calciuresis via stimulation of the basolateral CaSR in the thick ascending loop (TAL) of Henle (303, 385). These observations suggest that when the CaSRs of DCT cells are induced on both membranes simultaneously the effect produced via the apical side predominates. The role of the basolateral CaSR in the DCT/CNT could then be to counterregulate that of the apical one.

####### 4.8.1.3.1.2.7. parathyroid hormone.

Parathyroid hormone (PTH) has been found to decrease Na^+^-Cl^−^ cotransport by inducing phospholipase C (PLC) as well as ERK1/2 through activation of a basolaterally disposed G protein-coupled receptor (386, 387). This effect on NCC would thus predictively increase the Em of DCT cells and stimulate the luminal uptake of Ca2+ (via TRPV5) secondarily (387). In other studies, however, PTH has been shown to increase NCC phosphorylation (211) or to exert no consequence on the transport of Na+ (387, 388).

####### 4.8.1.3.1.2.8. ion conductance in dct cells.

The *E*_m_ and [Cl^−^]_i_ of DCT cells are important determinants of NCC activity. They are also affected by NCC itself but most notably by a heteromeric K^+^ channel called Kir4.1-Kir5.1 that is expressed in DCT1 and DCT2 (389–392) and by CLCKB that is expressed throughout the distal nephron (390, 393, 394). These channels consist of basolaterally disposed outward conductive pathways and appear to be electrogenically coupled in that the movement of ions through one channel drives the movement of ions through the other channel in the same direction (252, 392, 395, 396). The Kir4.1-Kir5.1 heterodimer has also been said to act as a sensor of [K^+^]_o_ in DCT cells (391, 392).

There are several lines of evidence to suggest that an increase in the Em of DCT1/DCT2 cells coupled to a decrease in [Cl^−^]_i_ causes NCC to undergo phosphorylation and increase in abundance (175, 179, 252, 347, 392, 395, 397, 398). This electrochemical trait (referred to here as the “A” trait) can be acquired when *1*) serum [K+] is reduced through a low-K+ diet or renal K+ wasting or *2*) the outward conductance of K^+^ is enhanced through overexpression of homomeric Kir4.1 (399). Under either condition, K+ is forced out of the cells such that Cl^−^ follows behind and [Cl^−^]_i_ decreases (FIGURE 10*B*) (252, 392, 395, 396, 399, 400). While, importantly, a decrease in NCC activity causes [K^+^]_i_ to increase initially, it can also cause serum [K^+^] to decrease and [K^+^]_i_ to fall below normal values as a result.

Many regulatory steps have been found to underlie the effect of the “A” trait on NCC phosphorylation/abundance. They were identified by characterizing the phenotype of Kir4.1-null as well as K^+^-deprived mice and shown to include OSR1, WNK4, and KLHL3 phosphorylation as well as WNK body formation (245, 252, 255, 256, 395, 401–404). Based on the studies carried out, SPAK did not appear to play a substantial role in the cascade involved (252, 401), and activation of WNK4 was attributed to a decrease in [Cl^−^]_i_ (252, 256, 259, 260, 392, 403). As for the mechanisms that account for the dependence of NCC activity on Em, they do not appear to have been formally explored (405, 406).

There is also evidence to suggest that a decrease in the Em of DCT//DCT2 cells or an increase in [Cl^−^]_i_ causes NCC to undergo dephosphorylation (179, 212, 252, 260, 396, 407–410) and decrease in abundance under many circumstances. This other electrochemical trait (referred to here as the “I” trait) can be acquired when *1*) serum [K+] is increased through a high K+ load or drugs, *2*) the outward conductance of K^+^ is reduced through deletion of Kir4.1, *3*) [Cl^−^]_o_ is increased through incubation of cells/tissues in high-Cl^−^ medium, or *4*) the outward conductance of Cl^−^ is reduced through deletion of CLCKB or the use of Cl^−^ channel blockers (see FIGURE 10*B*) (252, 392).

As for the regulatory steps that underlie the effect of the “I” trait on NCC phosphorylation and abundance, they appear in many conditions (252, 260, 392, 393, 396, 411, 412) to include both SPAK and WNK phosphorylation (392, 411, 413). For the condition “high K^+^ load,” however, the steps involved are probably not the same. In particular, they could require PP3 and CaM rather than WNK (291, 410, 414, 415) and come with very rapid dephosphorylation of NCC but no change in carrier abundance (212, 347, 396, 407, 408, 416). Given that PP3 should decrease WNK abundance by activating KLHL3, one would have to presume that this effect is counteracted in some way by the high K^+^ load.

####### 4.8.1.3.1.2.9. the na^+^-k^+^-atpase.

In the DCT, a decrease in the activity of the Na^+^-K^+^-ATPase on the basolateral side comes with a decrease in that of NCC (325). It is relevant to mention that this enzyme is upregulated (in abundance) by a transcription factor called HNF1β, that its K^+^ transport site is supplied for by Kir4.1 (325), that it must associate with FXYD2 to be fully active, and that its transport function is of course ATP dependent.

###### 4.8.1.3.2. Systemic roles of NCC activity in the DCT.

####### 4.8.1.3.2.1. ecfv and bp control.

####### 4.8.1.3.2.1.1. evidence for the role of ncc.

By allowing a variable and potentially important percentage of the ultrafiltered Na^+^ load to be reabsorbed by the kidney, NCC plays an important role in ECFV homeostasis and BP control (417). As reviewed in this section, there are numerous lines of direct and indirect evidence not only to support this assertion but also to suggest that the Na^+^-Cl^−^ cotransport mechanism is an essential player in the fine-tuning of salt reabsorption by the DCT.

One such line of evidence is, as mentioned, that NCC activity is upregulated by conditions that lead to ECFV contraction (such as dietary NaCl restriction and volume depletion) and by hormones that are recruited as a result of ECFV contraction (such as aldosterone, Ang II, vasopressin, and catecholamines) (345, 358, 364, 418). Of course, other DCT-based Na^+^-reabsorbing transport systems are upregulated by these conditions/hormones, but they do not allow for a response that can fully replace the contribution of NCC (see sect. 4.9).

Another line of evidence for the role of NCC in ECFV regulation is that hyperinsulinemic states are typically associated with systemic hypertension and with enhanced natriuresis in response to thiazides (370–374). It is worth mentioning once again in this regard that hyperinsulinemic animal models such as the obese Zucker rat exhibit enhanced phosphorylation of PI3K, WNK4, SPAK, and NCC (295, 296, 375). Whether there is a physiological purpose to the effect of insulin on ECFV is nonetheless unclear.

A third line of evidence is that a low-K^+^ diet, which corresponds to another NCC-activating factor, has been found to cause a thiazide-sensitive form of systemic hypertension (252, 296, 398, 419–422) and a high-K^+^ diet to protect against chronic BP elevation (421–424). It has also been hypothesized that drugs such as angiotensin-converting enzyme, Ang II receptor, and ENaC inhibitors could exert part of their action as BP-lowering agents by increasing serum [K^+^] through renal K^+^ retention (422). The physiological purpose behind the effect of a change in K^+^ diet on ECFV is discussed further below.

From the clinical standpoint, the importance of NCC in ECFV homeostasis is highlighted somewhat indirectly by the phenotype that accompanies the different types of inherited PHAII in humans (see sect. 4.9). Although this phenotype appears to be accounted for to a large extent by an increase in Na^+^-Cl^−^ cotransport activity along the DCT, it is typically one of high BP that is exquisitely sensitive to thiazides (425).

Genome-wide association studies between members of the WNK-SPAK-NCC pathway and BP traits in humans initially revealed promising evidence of linkage (322, 426–431). In certain populations, for instance, they led to the identification of polymorphisms that were associated with lower BP (NCC) or essential hypertension (NCC, SPAK, WNK1, or WNK4). However, it was found eventually that segregation between genotype and phenotype in these studies failed to reach robust statistical significance and that the polymorphisms identified had little or no impact on the natriuretic effect of thiazides in clinical trials (432–436).

####### 4.8.1.3.2.1.2. role of ncc in the circadian variation of bp and natriuresis.

Under a diurnal rhythm, aldosterone secretion, BP, and renal Na^+^ excretion are higher during the morning and afternoon than they are during the night, and they typically reach their lowest points a few hours before waking (437, 438). These fluctuations and those of a wide variety of physiological and biological processes are now known to be driven by circadian regulators including the so-called period (*PER*), cryptochrome (*CRY*), and clock (*CLOCK*) genes (439).

Studies conducted in mouse have shown that NCC expression, phosphorylation, or activity in kidney tissues or urinary exosomes was higher in active mice compared with inactive ones (438, 440–443) and that WNK1, SPAK, or OSR1 phosphorylation was also higher during the same period (440, 441). As one might have expected, the mineralocorticoid receptor (440, 443) and circadian genes such as *PER1* and *PER2* have been identified as active contributors to these observed differences (441, 442).

It is thus interesting that the timing of aldosterone secretion and BP was found to be aligned with the activity of the WNK1/SPAK-OSR1/NCC pathway under the influence of circadian genes. These observations would be consistent with the idea that NCC plays an important role in shaping the 24-h BP profile of normal individuals. The question that still comes to mind is why urinary Na^+^ excretion over a 24-h cycle would be higher in the morning and afternoon than at night if it were largely dependent upon the aldosterone/NCC pathway (437).

If NCC did hold significant relevance in the circadian regulation of ECFV, one could then postulate that inadequate nocturnal suppression of the cotransport mechanism in the context of certain conditions could prevent BP from dipping normally. Such conditions are known to include advanced age, chronic kidney disease, disrupted sleep patterns, several drugs, and so on (444). Under such circumstances, the administration of thiazides at bedtime could hypothetically restore the nondipping anomaly and decrease the mortality that comes with it. At the same time, this approach cannot be advocated as it stands, in that its usefulness has not been verified through clinical trials (445, 446).

Studies in humans have shown that NCC abundance (based on expression studies in urinary exosomes) tended to increase during the day, albeit rather modestly (447). These additional observations would thus indicate that the data gathered from rodent studies on the circadian rhythm of renal salt transport do not entirely apply to humans. At the same time, it would certainly be of interest to determine whether NCC, WNK4, and SPAK phosphorylation levels are also key to 24-h variations in human urinary exosomes.

####### 4.8.1.3.2.2. k^+^ homeostasis.

**FIGURE 11.**
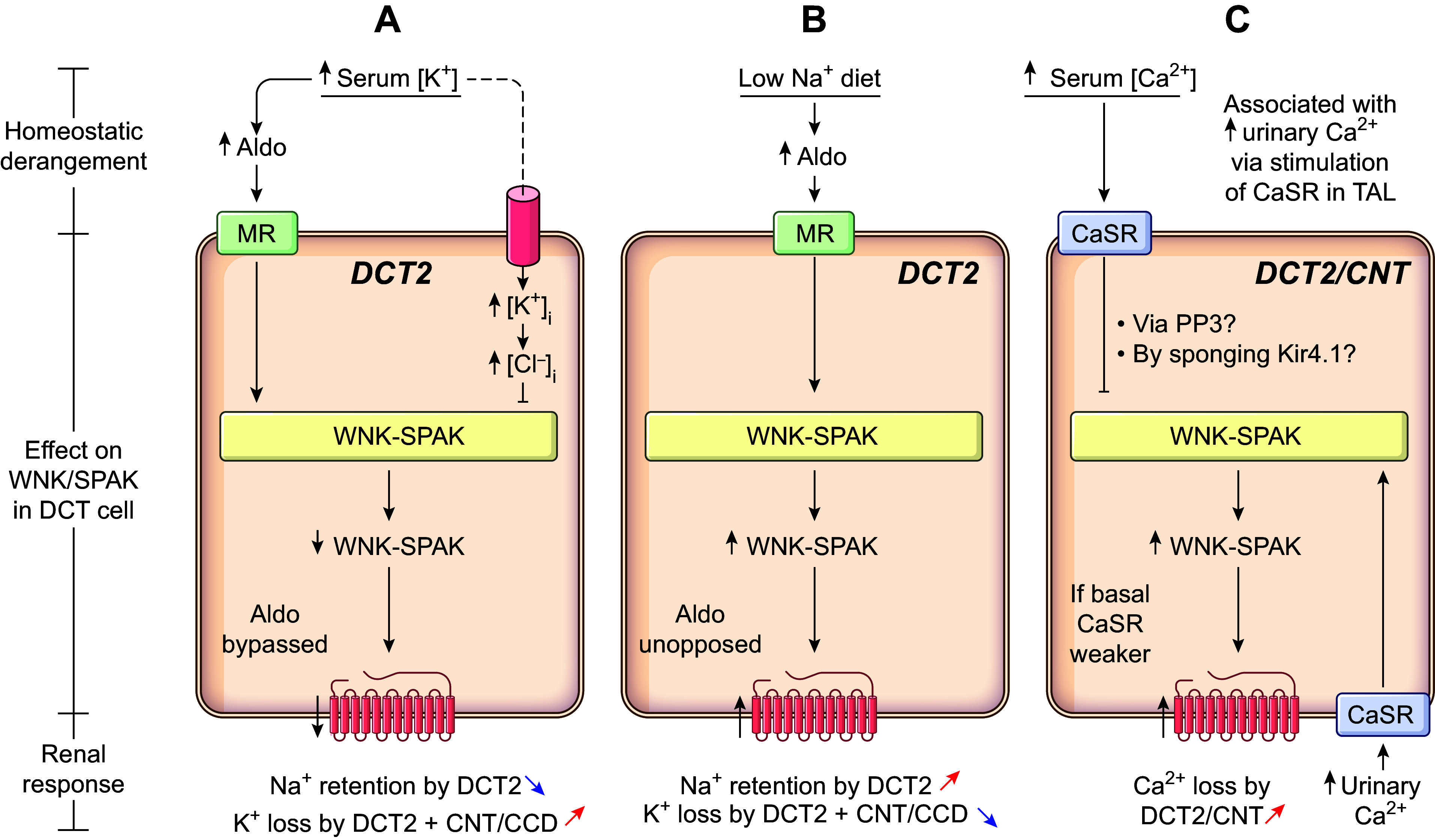
Homeostatic roles of the Na^+^-Cl^−^ cotransporter (NCC) in the distal convoluted tubule (DCT) based on 3 examples. *A*: hyperkalemia. NCC responds to a rise in serum [K^+^] by decreasing its activity to enhance Na^+^ delivery at the ENaC- and ROMK-expressing sites (252, 260, 412, 448). Na^+^ reabsorption should be minimally affected under such circumstances as it is assumed by another ion transport system. It is of note that a rise in serum [K^+^] should theoretically lead to activation of the WNK1/WNK4-SPAK/OSR-NCC pathway in DCT cells by increasing aldosterone production (46, 101, 339, 340). However, it leads in the end to inactivation of this pathway, as it also causes [Cl^−^]_i_ to rise (252, 260, 412, 448). *B*: Na^+^ restriction. NCC responds to a decrease in dietary Na^+^ by increasing its activity to enhance Na^+^ reabsorption (46, 101, 339, 340). K^+^ secretion should increase minimally under such circumstances, as Na^+^ delivery at the ENaC- and ROMK-expressing sites decreases. *C*: hypercalcemia. NCC could respond to a rise in serum [Ca^2+^] by increasing its activity to promote urinary Ca^2+^ loss. The CaSR would be likely involved in this process even if it has been shown to cause NCC activity to decrease when activated on the basolateral membrane of DCT cells (381). It is indeed also expressed on the apical membrane of DCT cells, and its activation at this other location has been shown to increase NCC activity (328, 382). Hypercalcemia would then trigger the apical response by causing Ca^2+^ reabsorption by the thick ascending loop (TAL) to decrease (303, 385). Downward arrow, minimized; upward arrow, maximized. MR, mineralocorticoid receptor. See glossary for additional abbreviations.

NCC has long been known to be involved in K^+^ homeostasis by impacting on K^+^ renal handling indirectly (65). As illustrated in part in FIGURE 11*A*, the main evidence pertaining to this role is that NCC phosphorylation and activity are upregulated by a low-K^+^ diet (where K^+^ retention is required) in both DCT1 and DCT2 and downregulated by a high-K^+^ load (where K^+^ wasting is required) (252, 260, 412, 448). Insulin could also exert a stimulatory effect on NCC to sustain its own anabolic role by minimizing renal K^+^ wasting (295, 296, 370).

It is logical that under a low-Na^+^ diet or during ECFV contraction the ensuing rise in serum aldosterone would stimulate NCC directly in DCT2. It would then indeed cause the transporter to maximize Na^+^ reabsorption by the kidney but to minimize K^+^ secretion in parallel by limiting Na^+^ delivery at the K^+^ secreting sites while ENaC is upregulated in DCT2 and CNT (see FIGURE 11*B*). It is also logical that under a high-Na^+^ diet or during ECFV expansion the ensuing decrease in serum aldosterone would exert the opposite effect on ion handling by causing NCC and ENaC to be downregulated.

When aldosterone production is driven by serum [K^+^], it is then illogical that NCC would be solely regulated by this hormone. For instance, an increase in serum aldosterone resulting from an increase in serum [K^+^] would have the effect (if unopposed) of stimulating NCC in DCT2 and leading not only to K^+^ retention but also to increased Na^+^ absorption. By contrast, a decrease in serum aldosterone resulting from a decrease in serum [K^+^] would have the effect (if also unopposed) of downregulating NCC and leading not only to K^+^ wasting but also to decreased Na^+^ absorption.

As indicated above, DCT cells are endowed with K^+^ sensing ability to regulate NCC activity, i.e., to decrease Na^+^-Cl^−^ cotransport when serum [K^+^] increases and to increase Na^+^-Cl^−^ cotransport when serum [K^+^] decreases (252, 260, 412, 448). If the stimulus for aldosterone production is a change in serum [K^+^], the impact of this hormone on NCC is thus overridden by the K^+^-sensing mechanism of DCT1 and DCT2 to ensure that renal K^+^ handing is compensatory and Na^+^ handling minimally affected (see FIGURE 11*A*) (6).

It is conceivably because of this sensing mechanism that aldosterone can be effectively involved in both K^+^ and ECFV homeostasis without compromising one role against the other. However, it is not clear that these roles are perfectly counterbalanced at all times, especially when they are chronically solicited. As already alluded to, long-term dietary K^+^ restriction does appear to generate an overactive NCC-like phenotype with high BP as if the effect of aldosterone suppression on NCC became eventually less important than that of the K^+^-sensing machinery.

####### 4.8.1.3.2.3. urinary dilution.

NCC plays an important role in urinary dilution through its presence along most cell types along the DCT. With the aid of the Na^+^-K^+^-ATPase pump and CLCKB, it allows urine osmolality to decrease from 150 mosmol/kgH_2_O after the macula densa to 100 mosmol/kgH_2_O in the late DCT2 (301). This difference might appear small but is substantial considering that the CCD ensures higher levels of water reabsorption than the medullary collecting duct in response to vasopressin (449).

The role of NCC in water dilution is suggested by a combination of observations and/or deductions. In particular, Na^+^-Cl^−^ cotransport is the main mechanism of Na^+^ uptake by DCT cells, it is upregulated by vasopressin through various signaling pathways (as described above), and its inhibition through the administration of thiazides has been associated with a propensity to develop water retention accompanied by hyponatremia in elderly women (325, 335, 387, 450, 451).

####### 4.8.1.3.2.4. ca^2+^ and mg^2+^ homeostasis.

NCC activity has also long been known to affect the renal handling of Ca^2+^ and Mg^2+^ (325, 335, 387, 451). However, whether this is an indication that the Na^+^-Cl^−^ cotransport mechanism plays an active homeostatic role in the balance of divalent cations is still currently unclear. In the case of Ca^2+^, this possibility should be kept in mind because of the effect of apical CaSR stimulation on NCC activity in the DCT (see illustration of this concept in FIGURE 11*C*) (328). In addition, CaSR activity could be itself sensitive to NCC-dependent changes in intracellular ion concentration.

#### 4.8.2. Roles in other tissues.

##### 4.8.2.1. introduction.

Although NCC is widely distributed and expressed at relatively high levels in a number of nonrenal cell types, its functional relevance outside of the DCT is still largely unknown and poorly understood. A few exceptions could nonetheless apply in this regard, as there are some indications or reasons to believe that the Na^+^-Cl^−^ cotransport mechanism does play a physiological role in certain tissues. Two examples are presented below.

##### 4.8.2.2. tissues of interest.

###### 4.8.2.2.1. Bone.

A number of observational studies have allowed showing that long-term treatment with thiazides and Gitelman syndrome both protect against bone mass loss. Based on one of their recent metanalyses, Cheng et al. (452) concluded that thiazides did appear to offer this advantage in human but that studies of higher quality would be required to establish a more robust association between inhibition of NCC and markers of bone health.

Earlier studies have also shown that NCC is expressed at the surface of osteoblasts and that its inhibition at this location could explain why thiazides act as bone-preserving agents (453–455). In two such studies, it was found in particular that inactivation of the Na^+^-Cl^−^ cotransport mechanism in cultured osteoblasts led these cells to differentiate and coalesce into mineralized nodules (454, 455). In one of the studies, NCC inactivation in mouse also led diaphyseal bone mineral density, trabecular bone volume, and cortical wall thickness to increase (455).

NCC inhibition could produce its effect on bone cells by activating Ca^2+^-dependent transport systems as it does in the renal epithelium and by promoting osteoid mineralization as a result (453). NCC inhibition could also lead to stimulation of osteoblastogenesis if the activity or expression of certain differentiating factors (such as those of the Wnt-β-catenin-Runx2 or FAK-JNK-c-jun pathways) increased in response to lower [Cl^−^]_i_ or higher *E*_m_.

###### 4.8.2.2.2. Small intestine.

NCC is expressed in the duodenum, jejunum, and ileum but at relatively low levels based on RNA detection methods. It acts in these tissues as an apical transporter (456), as it does in DCT and winter flounder bladder (28), and is present in the villous aspect of the intestinal epithelium. Electrophysiological studies have also shown that addition of HCTZ to the luminal side of isolated intestinal segments caused the *E*_m_ of enterocytes to increase and transepithelial potential to decrease (456). Together, these observations indicate that the gut is capable of active Na^+^-Cl^−^ cotransport.

NCC has also been found to affect Ca^2+^ transport in the small intestine as it was shown to do in DCT. For instance, HCTZ was seen to elicit TRPV5- and TRPV6-related currents in an intestinal cell line (456) and NCC inactivation to increase Ca^2+^ absorption by the duodenum along with TRPV6, calbindin-D9K, and PMCA1b expression levels in a mouse model (454). By stimulating both the absorption of Ca^2+^ by the duodenum and the DCT, thiazides could thus exert a positive effect on Ca^2+^ balance and contribute to bone mass preservation through this other mechanism.

NCC inactivation in Gitelman syndrome does not lead to hypercalcemia even if it causes Ca^2+^ absorption by the kidney and duodenum to increase (457). It would thus be expected to exert the opposite effect in the jejunum, ileum, and/or colon. As it stands, this possibility has not been explored directly or by examining the consequence of reduced Na^+^-Cl^−^ cotransport on Ca^2+^ excretion in 24-h stool collections. As such, there is no evidence to indicate that thiazides could protect against bone mass loss through a systemic mechanism.

### 4.9. Pathology of NCC

#### 4.9.1. Classification.

Although the DCT only reabsorbs 5% of the ultrafiltered Na^+^ load, small changes in this percentage can translate into substantial clinical repercussions by altering ECFV and the transport of other ions by and beyond this nephron segment. For instance, Gitelman syndrome is characterized by low-normal BP in many affected individuals and PHAII by high BP in most eventually (458). Along the same line, systemic hypertension is often very sensitive to NCC inhibitors when it develops during or after the middle age (10, 459).

In sect. 4.9.2, the various disease states or drugs that have been linked to clinically relevant changes in NCC activity are reviewed (see list in TABLE 2). They are classified as either primary forms of NCC dysfunction (where Na^+^-Cl^−^ cotransport is perturbed because of a defect in NCC) or secondary forms of dysfunction (where Na^+^-Cl^−^ cotransport is perturbed because of a defect in a signaling intermediate or another ion transport system). The proteins at fault include those of the KLHL3-WNK1/WNK4-SPAK/OSR1 pathway, CLCKB, Kir4.1-Kir5.1, and the Na^+^-K^+^-ATPase (along with ATP1A, FXYD2, and HNF1β).

**Table 2.** Disease states or drugs that have been linked to clinically relevant changes in NCC activity

Primary	Inherited	NCC	LoFM in NCC (GS) LoFP in NCC?	GoFP in NCC?
	Acquired	NCC	Thiazides	
Secondary	Inherited	KLHL3-WNK-SPAK/OSR1	LoFM in WNK1 LoFP in WNK-SPAK/OSR1?	Gene unknown (PHAIIA) GoFM in WNK4 (PHAIIB) GoFM in WNK1 (PHAIIC) LoFM in KLHL3 (PHAIID) LoFM in CUL3 (PHAIIE) GoFP in WNK/SPAK/OSR1?
		Cl^−^ channels	LoFM in CLCKB (BSIII) LoFM in BSND (BSIVA) LoFM in CLCKA + CLCKB (BSIVB)	
		K^+^ channels	LoFM in Kir4.1 (ES) LoFM in Kir5.1	
		Na^+^-K^+^-ATPase	LoFM in ATP1A1 LoFM in FXYD2 LoFM in HNF1β LoFM in mitochondrial genes	
	Acquired	KLHL3-WNK-SPAK/OSR1		Calcineurin inhibitors SGLT2 inhibitors

BS, Bartter syndrome; ES, EAST/SeSAME syndrome; GoFM, gain-of-function mutation; GoFP, gain-of-function polymorphism; GS, Gitelman syndrome; LoFM, loss-of-function mutation; LoFP, loss-of-function polymorphism; NCC, Na^+^-Cl^−^ cotransporter; PHA, pseudohypoaldosteronism. See glossary for additional abbreviations.

The mechanisms that account for the abnormalities observed in several of the primary and secondary forms of NCC dysfunction are discussed succinctly here as they have been the subject of many comments already and can be deduced to some extent through the aid of FIGURE 10. In sect. 4.9.2, we also place focus on the human disorders given that the various animal models of “DCTopathies” have provided relatively limited insight into the clinical repercussions of the genetic defects at play.

#### 4.9.2. Primary dysfunction or NCC-related DCTopathy.

##### 4.9.2.1. conditions associated with a primary decrease in ncc activity.

###### 4.9.2.1.1. Inherited NCC dysfunction.

Gitelman syndrome is the name that is given to the primary form of inherited NCC dysfunction. It was first described by Dr. Hillel Jonathan Gitelman in 1966 (460), linked to the gene responsible 30 years later, and found to be recessively transmitted (67, 461, 462). It corresponds to the most common form of hereditary tubulopathy occurring at a prevalence of 1 per 50,000 individuals from a carrier pool of 500/50,000 (463, 464).

The tubulopathy of Gitelman syndrome is characterized *1*) by Na^+^, K^+^, Mg^2+^, and H^+^ renal wasting that leads (respectively) to ECFV contraction with hyperreninemic hyperaldosteronism, hypokalemia, hypomagnesemia, and metabolic alkalosis and *2*) by normocalcemic hypocalciuria (465, 466). Even though hypercalcemia is generally not a feature of this syndrome (457), individuals can also develop ectopic calcifications in the form of chondrocalcinosis or sclerochoroidal calcifications perhaps related to the functional dependence of certain pyrophosphatases on Mg^2+^ ions (466, 467).

Another manifestation that has been associated with Gitelman syndrome is a tendency toward impaired insulin sensitivity and secretion (468–470) that can translate into a prediabetic state in as much as 15% of affected individuals (470). The mechanisms at play are still unknown but could involve the electrolyte disorders that come with this syndrome, as both hypokalemia and hypomagnesemia have been associated with markers of glucose intolerance (471, 472). Additional or alternative explanations could have to do with the potential loss of NCC activity in the β cells of Langerhans islets and/or in insulin-sensitive tissues.

Gitelman syndrome is usually identified during or after adolescence in the course of a routine blood workup or of screening tests (473, 474). It is most commonly asymptomatic or only mildly symptomatic but can be more severe in certain individuals and occur at an earlier age (475, 476). When it is symptomatic, manifestations are then due to ECFV contraction, hypokalemia, or hypomagnesemia, and they can include a failure to thrive (463).

It should be mentioned here that although this DCTopathy is considered to be a recessive disorder, the carrier state is not completely asymptomatic. In particular, Blanchard et al. (470) found that heterozygote loss-of-function mutations in SLC12A3 were associated in 81 individuals with a subtle but clear intermediate phenotype. Compared with healthy volunteers, it came indeed with modest but frank insulin resistance, slightly higher serum aldosterone and [Ca^2+^] levels, as well as slightly lower serum PTH and urinary [Ca^2+^] levels.

Gitelman syndrome can be difficult to distinguish from the other inherited forms of DCTopathies (see below) or cryptic diuretic abuse and should therefore be confirmed through DNA testing when it is suspected (477). Although most patients with this syndrome should probably be treated with tolerable doses of Cl^−^-based K^+^ and Mg^2+^ supplements, those who are moderately to severely symptomatic should also be considered for treatment with angiotensin-converting enzyme or Ang II receptor inhibitors, potassium-sparing diuretics, and nonsteroidal anti-inflammatory drugs (463, 478–481).

Finally, and as said above, genome-wide association studies have shown that loss-of-function polymorphisms in NCC could be associated with altered BP in the general population (430). However, these studies were overall statistically inconclusive and only allowed identification of a few genetic variants of potential clinical relevance (432–435). NCC could still be an important determinant of BP variation in the general population but essentially as a polygene rather than as a monogene.

###### 4.9.2.1.2. Acquired dysfunction.

The most common side effect of thiazides is a DCTopathy that resembles that of Gitelman syndrome. Yet one must remember that the acquired and the genetic disorder are probably not interchangeable models of NCC dysfunction. In the former type NCC activity is intermittently and variably decreased among the cells where it is expressed, but in the latter type it is constantly and ubiquitously abolished. A question that comes to mind in this regard is whether thiazides could reach sufficiently high concentrations at the NCC-expressing sites of osteoblasts or distal intestinal cells to exert a strong inhibitory effect on the Na^+^-Cl^−^ cotransport mechanism.

##### 4.9.2.2. conditions associated with a primary increase in ncc activity.

To this date, there have been no reports of inherited human disorders in which NCC was found to harbor a gain-of-function mutation. There have been no reports either of animal models that were engineered to express high levels of Na^+^-Cl^−^ cotransport by altering the gene itself. As was hinted, however, presumed gain-of-function polymorphisms in NCC have been associated with higher BP and hypercalciuria in certain populations (320–322).

#### 4.9.3. Secondary dysfunction.

##### 4.9.3.1. klhl3-wnk-spak/osr1 pathway.

###### 4.9.3.1.1. Conditions associated with a secondary (WNK-dependent) increase in NCC activity.

####### 4.9.3.1.1.1. inherited dysfunction.

NCC can become overactive in the setting of monogenic defects. The phenotype seen in this context has been referred to as PHAII, meaning that it is characterized by resistance to the action of aldosterone but is not associated with renal salt wasting and ECFV contraction as in PHAI. It is also named familial hyperkalemic hypertension or Gordon syndrome after the physician (Richard A. Gordon) who singularized this presentation in 1970 by characterizing Australian families affected by a PHAII-like phenotype (482).

Five forms of PHAII among >80 families have now been described (482). One such form has been linked to locus 1q31-q42 (PHAIIA) and the four others to specific genes, i.e., WNK4 (PHAIIB), WNK1 (PHAIIC), KLHL3 (PHAIID), and CUL3 (PHAIIE). Whereas inheritance is dominant in PHAIIA, PHAIIB, PHAIIC, and PHAIIE, it can be both dominant and recessive in PHAIID (425). Mutations in KLHL3 are most commonly the cause of PHAII, and mutations in CUL3 are often de novo (268, 483).

As mentioned above, the known mutations in PHAII all act by increasing WNK expression in the DCT such that they stimulate SPAK/OSR1 activity and Na^+^-Cl^−^ cotransport secondarily. Those in WNK1 and WNK4 are gain of function as they prevent (in most cases) the enzymes from interacting with the KLHL3-CUL3 complex and undergoing degradation by the proteasome (222, 431). Those in KLHL3 and CUL3 are, by contrast, loss of function as they prevent the complex from functioning adequately (269).

The typical manifestations of PHAII are hyperkalemia, hyperchloremic metabolic acidosis, hypercalciuria, high BP, suppressed serum renin levels, and normal or increased serum aldosterone levels (484, 485). High BP is due in part to increased Na^+^ reabsorption by the DCT, the electrolyte disorder to the ensuing decrease in Na^+^ delivery at the K^+^-secreting sites of the nephron, renin suppression to ECFV expansion, and lack of aldosterone suppression to increased serum [K^+^] (284, 483). For reasons that have already been discussed above, hypermagnesemia is otherwise not a manifestation of PHAII.

The clinical presentation and age at diagnosis are quite varied among the PHAII subtypes and affected family members in a given pedigree (425). It is more severe and early onset in PHAIIE (CUL3) and in recessive PHAIID (KLHL3). Hyperkalemia is the most common finding, and high BP tends to become apparent only during adulthood (268). Other manifestations, probably in relation with the accompanying electrolyte and acid base disorders, have also been described in the more severe or early forms of PHAII. They include hypocalcemia, kidney stones, decreased bone mineral density, dental abnormalities, infertility, and failure to thrive (486–488).

In PHAII, high BP, hyperkalemia, and metabolic acidosis are usually highly responsive to thiazides and should thus be treated first line through the use of such drugs (486). One must not forget that BP management and diuretics can lead to adverse fetal outcome (425, 489–491). As such, pregnant women affected by PHAII should be referred to specialized obstetric clinics for proper adjustment of antihypertensive therapy and close monitoring of BP as well as serum electrolytes.

####### 4.9.3.1.1.2. acquired dysfunction.

As stated in above sections of this review, calcineurin inhibitors often lead to a PHAII-like phenotype and appear to do so (at least in part) by increasing NCC activity through activation of the PP3-KLHL3-WNK-SPAK signaling cascade (291, 492). However, PP3 is known to affect the activity of several other ion transport systems in many different cell types (493–495). One of these transport systems, a K^+^-ATP channel (Kir6.2/SUR2 or KCNJ11/ABCC9), is expressed in striated muscle cells, where it plays an important role in extracellular K^+^ buffering (496, 497).

###### 4.9.3.1.2. Conditions associated with a secondary (WNK-dependent) decrease in NCC activity.

Missense mutations in the Ct of WNK1 have been linked to a salt-losing form of hypermagnesemic tubulopathy in three individuals (324). One such individual harbored a de novo substitution (I1172M) in exon 16 and the two others (father and son) an inherited substitution (S2047N) in exon 24. Interestingly, the three individuals also harbored a heterozygote loss-of-function mutation in NCC, and their family members were not symptomatic unless they carried both the WNK1 and NCC mutation. Furthermore, HEK-293 cells coexpressing WNK1_I1172M_ and NCC_WT_ were shown to exhibit decreased carrier expression at the cell surface.

##### 4.9.3.2. ion transporters associated with a secondary decrease in ncc activity.

###### 4.9.3.2.1. CLCKB.

As discussed in sect. 4.8, the serosal step of Cl^−^ reabsorption in the DCT is ensured by CLCKB (see FIGURE 9) (498) and is a limiting factor for the luminal step of NaCl reabsorption by NCC. For this reason, a decrease in Cl^−^ channel activity leads not only to lowered Na^+^-Cl^−^ cotransport but also to increased [Cl^−^]_i_ such that *E*_m_ should rise and WNK activity decline. Of note, this Cl^−^ channel is expressed in other nephron segments such as the TAL (499).

Biallelic loss-of-function mutations in CLCKB, in BSND (which is essential to the processing of CLCKB), and in both CLCKB and CLCKA have been linked to Bartter syndrome III (BSIII), BSIVA, and BSIVB, respectively (499, 500). They also lead to renal salt wasting but are much less common in the population than loss-of-function mutations in NCC (501). Overall, the Bartter syndromes, which include BSI, BSII, and BSV in addition to BSIII and BSIV, occur at a prevalence of 1 per 1,000,000 (500, 502).

CLCKB- or BSND-related tubulopathies share many phenotypic traits with the NCC-related tubulopathy. In particular, they have been associated in many cases with hypocalciuria as well as with Na^+^, K^+^, Mg^2+^, and H^+^ renal wasting (503, 504). In contrast to Gitelman syndrome, however, they typically present with hyperprostanglandinemia, and, in contrast to BSI and BSII, they rarely present with substantial hypercalciuria (505–507). The mechanisms underlying the Ca^2+^ handling phenotype in these tubulopathies are not completely understood. They could be related to the consequences of a defect in Cl^−^ conductance on NCC activity (and distal NaCl delivery by the same token), on *E*_m_, or on WNK signaling.

Like Gitelman syndrome, BSIII is usually identified at a more advanced age (during childhood or adolescence) and is typically (albeit not always) associated with a mild form of tubulopathy (508, 509). On the other hand, BSIVA and BSIVB come with a more severe renal salt wasting that leads to polyuria and can cause polyhydramnios with premature birth if present antenatally (510, 511). Sensorineural hearing loss is also a common manifestation of BSIVA and BSIVB (512). The treatments recommended for these disorders are the same as for Gitelman syndrome (506).

###### 4.9.3.2.2. K^+^ channels.

####### 4.9.3.2.2.1. generalities.

In the renal epithelium, Kir4.1 and Kir5.1 are expressed as a heterodimeric structure in the basolateral membrane of DCT (see FIGURE 9) and CCD cells, where they play a role in *E*_m_ regulation and ensure K^+^ recycling to sustain Na^+^-K^+^-ATPase pump activity (513, 514). These channels are thus again a limiting factor for both the serosal and luminal steps of Na^+^ reabsorption in the DCT. Kir5.1 is also known to be associated with other types of K^+^ channels in other nephron segments (391, 515).

All in all, the consequences of a decrease in Kir activity in the DCT are as follows: *1*) increase in [K^+^]_i_ and in [Cl^−^]_i_ secondarily, *2*) decrease in the activity of Na^+^-K^+^-ATPase and of NCC secondarily, and *3*) decrease in *E*_m_ resulting from the decrease in K^+^ channel and pump activity. One would also expect the WNK-SPAK/OSR1 signaling pathway to be downregulated under such circumstances.

####### 4.9.3.2.2.2. k^+^ transport systems.

####### 4.9.3.2.2.2.1. kir4.1.

Biallelic loss-of-function mutations in Kir4.1 have been linked to a syndrome that is referred to by two different acronyms, i.e., EAST syndrome (epilepsy, ataxia, sensorineural deafness, and tubulopathy) and SeSAME syndrome (seizures, sensorineural deafness, ataxia, mental retardation, and electrolyte imbalance). The tubulopathy or electrolyte imbalance component of this syndrome is also one of renal salt wasting (406, 516). EAST/SeSAME syndrome has been described thus far in only a few families (517, 518).

The Kir4.1-related tubulopathy shares many phenotypic traits with the NCC-related tubulopathy (516). In particular, it presents in many cases with hypocalciuria as well as with Na^+^, K^+^, Mg^2+^, and H^+^ renal wasting. Interestingly, hypocalciuria is seen even if the *E*_m_ of DCT cells in EAST/SeSAME syndrome is much lower than in Gitelman syndrome, confirming that Ca^2+^ handling in either of these tubulopathies cannot be solely accounted for by a change in charge. In the Kir4.1-related one, it could still result from the defect in K^+^ conductance at the basal membrane of DCT1 and DCT2, as [Cl^−^]_i_ would be expected to increase under such circumstances and cause WNK signaling and/or NCC activity to decrease secondarily.

Besides those implied by the acronyms, the manifestations that can develop in association with biallelic loss-of-function mutations in Kir4.1 are as follows: diffuse parenchymal lesions of the kidney, nephrocalcinosis, miscellaneous syndromic features, delayed psychomotor development, pyramidal/extrapyramidal deficits, and thrombocytosis (516, 518). Why nephrocalcinosis has been observed in some cases is puzzling given the hypocalciuric phenotype.

EAST/SeSAME syndrome is usually identified during early childhood and comes with decreased life expectancy (518, 519). However, variability in severity and clinical manifestations has been described among unrelated individuals and within the same family (516, 520). The tubulopathy can also fluctuate in intensity over time (521). It should be treated otherwise through supportive measures as the tubulopathy of Gitelman syndrome.

####### 4.9.3.2.2.2.2. kir5.1.

Biallelic loss-of-function mutations in Kir5.1 have been found to cause a hypocalciuric (at times normocalciuric or even hypercalciuric) form of renal salt wasting with hypokalemia and deafness as in EAST/SeSAME syndrome (520, 522). However, many of the affected individuals suffer from renal tubular acidosis instead of metabolic alkalosis (399, 520, 522). This other trait is likely to be accounted for by the inactivation of Kir5.1 in the proximal tubule where the channel is normally expressed and associates with Kir4.2 to sustain $\mathrm{HC}O_{3}^{-}$ reabsorption and $NH_{4}^{+}$ secretion (523). It is also of note that the Kir5.1-related tubulopathy does not come with ataxia and epilepsy, in contrast to EAST/SeSAME syndrome.

###### 4.9.3.2.3. Na^+^-K^+^-ATPase.

####### 4.9.3.2.3.1. generalities.

The Na^+^-K^+^-ATPase is expressed on the basolateral membrane of the renal epithelium, where it is composed of the α1 ATP1A1 and β1 ATP1B1 subunits in all nephron segments and of the γ FXYD2 subunit in the PT, medullary TAL, DCT, and CNT (see FIGURE 9) (315, 524). The α1-β1-FXYD2 heterotrimer is particularly abundant in DCT cells, where its role is to ensure the serosal step of Na^+^ reabsorption and contribute to *E*_m_ regulation (525). The consequence of a decrease in Na^+^-K^+^-ATPase activity is an increase in [K^+^]_i_ and in [Cl^−^]_i_ secondarily as well as a decrease in *E*_m_ and in NCC activity. It would thus resemble that seen in Kir4.1-inactivated DCT cells.

There are four important settings in which Na^+^-K^+^-ATPase activity is expected to decrease in the DCT. They are as follows: *1*) biallelic loss-of-function mutations in Kir4.1, *2*) mono- or biallelic loss-of-function mutations in FXYD2, *3*) mono- or biallelic loss-of-function mutations in HNF1β, and *4*) a decrease in [ATP]_i_ resulting from mitochondrial disorders. The FXYD2-, HNF1β-, and ATP-related tubulopathies are the subject of further discussions below.

####### 4.9.3.2.3.2. related tubulopathies.

####### 4.9.3.2.3.2.1. fxyd2.

FXYD2 affects the functional and structural properties of α1 ATP1A1, i.e., causes this subunit to exhibit lower affinities for both Na^+^ and K^+^, higher affinity for ATP, and greater overall structural stability (526–530). It has been identified as a disease-causing gene in only three (apparently related) families through the monoallelic G41R mutation (531–533). This defect was also found to cause misrouting of the γ-subunit and to be associated with lower levels of Na^+^-K^+^-ATPase activity at the cell surface (534).

The phenotype observed in affected individuals was again one of normocalcemic hypocalciuria (as seen in the Kir4.1 tubulopathy) and one also of renal salt wasting (of Mg^2+^ most commonly and of K^+^ along with Na^+^ in some cases) that was found to vary in severity and age of onset (501, 532, 535). It was also accompanied by chondrocalcinosis in many of the reported individuals (532, 535). A concomitant defect in electrolyte handling by the medullary TAL could play a contributory role owing to the presence of FXYD2 in this nephron segment (536, 537).

####### 4.9.3.2.3.2.2. atp1a1

Heterozygous, de novo loss-of-function mutations in ATP1A1 were recently described in three unrelated children (538). They were found to be associated with *1*) severe and refractory renal magnesium wasting and seizures, *2*) episodes of hyperkalemia, *3*) metabolic alkalosis, and *4*) global psychomotor developmental delay (538, 539). These mutations were also shown to ablate pump activity and cause *E*_m_ to decrease in heterologous expression systems (538). The renal phenotype seen was thus quite reminiscent of that described in the FXYD2-related tubulopathy.

####### 4.9.3.2.3.2.3. hnf1β.

HNF1β (which stands for hepatic nuclear factor 1β) is a regulated transcription factor that is expressed in developing nephrons, in the branching ureteric bud, and throughout most of the mature renal epithelium (540–543). It plays an important developmental role in the kidney and upregulates the transcriptional activity of many ion transport systems including FXYD2, the CaSR, NKCC2, and urate transporters in the proximal tubule (544–547).

Monoallelic loss-of-function mutations in HNF1β have also been associated with hypocalciuria as well as various degrees of Na^+^, K^+^, Mg^2+^, and H^+^ renal wasting (548, 554). As this presentation is similar to that seen in the Kir4.1-, FXYD2-, and ATP1A1-related tubulopathies, it can probably be accounted for to some degree by a decrease in NCC activity and/or WNK signaling in the DCT. However, it is at times associated with renal wasting of other ions such that it is then likely to occur through ion transport defects in other nephron segments.

Monoallelic loss-of-function mutations in HNF1β have also been associated with several abnormalities of variable penetrance such as CAKUT, renal cysts, diabetes [mature-onset diabetes of the young type 5 (MODY-5)], elevated liver enzymes, neurological/cognitive/psychiatric disorders, delayed psychomotor development, and miscellaneous syndromic features (549). In 50% of cases, the genetic defect at play is a deletion of several genes within the 17q12 HNF1β-containing locus and could be then more commonly associated with heftier neurological/cognitive/psychiatric manifestations (548, 550).

####### 4.9.3.2.3.2.4. mitochondriopathy.

DCT cells must rely on a large contingent of mitochondria to ensure their transport functions (551). It is thus not surprising that mitochondrial disorders would be found eventually to be a cause of DCTopathy in certain cases. Wilson et al. (552) were the first research group to link a Gitelman-like form of tubulopathy to a mitochondrial tRNA gene (MT-TI; tRNA-iso) in a large family. Later on, an analogous phenotype was linked to the same MT-TI gene in four families and to another mitochondrial tRNA gene (MT-TF; tRNA-leu) in nine additional families (553).

A few additional observations in regard to the mitochondrial defects uncovered in these families appear important to mention. In one study, for instance, fibroblasts isolated from affected family members showed decreased mitochondrial function and heterologously expressed NCC_WT_ was seen to exhibit decreased phosphorylation in response to complex IV inhibition (553). Additionally, the MT-TF mutations identified were typically associated with chronic renal failure, and both the DCTopathy-causing MT-TI and MT-TF mutations had not been previously associated with mitochondriopathies.

## 5. CONCLUSIONS AND PERSPECTIVES

In this review, we have shown that NCC plays a critical role in the normal operation of the DCT and that it contributes to ECFV maintenance, BP control, K^+^ homeostasis, and urinary dilution. We have also described the mechanisms of carrier regulation, the structural determinants of ion translocation by this protein, the overall portrait of ion handling by the middle portion of the distal nephron, and the pathophysiology of various DCTopathies in the light of recent advances and hypothesis-driven considerations.

There are still various challenges that remain to be faced and research undertakings that are worth deploying in the field of Na^+^-Cl^−^ cotransport. One such challenge would be to determine whether NCC plays an important role in bone formation through its expression in osteoblasts. Another challenge would be to characterize the structural mechanisms of ion translocation and carrier regulation under various challenges to determine whether ion binding is ordered and whether transport stoichiometry is static at all times.

Since it was uncovered 50 years ago in the bladder of winter flounder, the Na^+^-Cl^−^ cotransport mechanism has been the subject of thousands of publications and has thus prompted tremendous interest in the field of transport physiology. It has also elicited indirect attention in many other fields such as those of human genetics, developmental neurobiology, and sensorineural hearing loss. It would perhaps gain in notoriety even more if it were amenable to tissue-targeted inhibition for the prevention of osteoporosis or the treatment of other diseases in which it plays an important pathophysiological role.

## GLOSSARY

ABCC: ATP binding cassette (ABC) transporter subfamily C
AKT: Protein kinase B
BSND: Barttin CLCK accessory subunit
CaB: Ca^2+^ binding protein
CALB: Calbindin
CaM: Calmodulin
CaMK: CaM-dependent protein kinase
CaSR: Ca^2+^-sensing receptor
CLCK: Chloride channel
CLDN: Claudin
CNNM: Cyclin M
CUL: Cullin
ENaC: Epithelial Na^+^ channel
FAK: Focal adhesion kinase
HNF1β: Hepatocyte nuclear transcription factor 1β
*J*
: Flux rate
JNK: c-Jun NH_2_-terminal kinase
KCNJ: K^+^ inwardly rectifying channel subfamily J
*K*_i_
: Inhibition constant
Kir: K^+^ channel inward-rectifying
KLHL3: Kelch Like Family Member 3
*K*_m_
: Ion constant (apparent affinity)
Kv: Voltage-sensitive K^+^ channel
MgBP: Mg^2+^ binding protein
NCX: Na^+^/Ca^2+^ exchanger
Nedd: Neural precursor cell expressed developmentally downregulated
NHE: Na^+^/H^+^ exchanger
OSR1: Oxidative stress-responsive kinase 1
PI3K: Phosphatidylinositol 3-kinase
PKA: Protein kinase A
PKC: Protein kinase C
PLC: Phospholipase C
PMCA: Plasma membrane Ca^2+^ ATPase
PP: Protein phosphatase
PT: Proximal tubule
ROMK: Renal outer medullary potassium channel
Runx: Runt-related transcription factor
SGK: Serum and glucocorticoid-regulated kinase
SLC: Solute carrier
SORL1: Sortilin-related receptor 1
SPAK: STE20/SPS1-related proline/alanine-rich kinase
SUR: Sulfonylurea receptor
TRPM: Transient receptor potential of the melastatin type
TRPV: Transient receptor potential of the vanilloid type
*V*_0_
: Transport rates in the absence of substrate
*V*_max_
: Maximal transport rates
WNK: With no lysine kinase
Wnt: Wingless/integrated

## GRANTS

This work was funded by the Kidney Foundation of Canada and the Canadian Institute of Health Research.

## DISCLOSURES

No conflicts of interest, financial or otherwise, are declared by the authors.

## AUTHOR CONTRIBUTIONS

A.V.R., A.P.G., and P.I. conceived and designed research; A.V.R., T.R.N.-B., S.S., N.A.D.B., M.A.M.G., S.V.S., L.H., A.P.G., and P.I. analyzed data; A.V.R., T.R.N.-B., S.S., N.A.D.B., M.A.M.G., S.V.S., L.H., A.P.G., and P.I. interpreted results of experiments; A.V.R. and P.I. prepared figures; A.V.R., A.P.G., and P.I. drafted manuscript; A.V.R., T.R.N.-B., S.S., N.A.D.B., M.A.M.G., S.V.S., M.J.F., L.H., A.P.G., and P.I., edited and revised manuscript; A.V.R., T.R.N.-B., S.S., N.A.D.B., M.A.M.G., S.V.S., M.J.F., L.H., A.P.G., and P.I. approved final version of manuscript.
